# Early Post-trauma Interventions in Organizations: A Scoping Review

**DOI:** 10.3389/fpsyg.2020.01176

**Published:** 2020-06-25

**Authors:** Matt T. Richins, Louis Gauntlett, Noreen Tehrani, Ian Hesketh, Dale Weston, Holly Carter, Richard Amlôt

**Affiliations:** ^1^Behavioural Science Team, Emergency Response Department Science and Technology, Health Protection and Medical Directorate, Public Health England, London, United Kingdom; ^2^Crisis, Disaster, and Trauma Section, British Psychological Society (Member of Standing Committee EFPA), Leicester, United Kingdom; ^3^Alliance Manchester Business School, University of Manchester, Manchester, United Kingdom

**Keywords:** early interventions, trauma, PTSD, emergency services, organizations

## Abstract

**Background:**

In some organizations, traumatic events *via* direct or indirect exposure are routine experiences. The National Institute for Health and Care Excellence reviews (2005; 2018) of post-traumatic stress disorder management in primary and secondary care did not address early interventions for trauma within emergency response organizations.

**Aims:**

This scoping review was designed to identify research which evaluates the use of early interventions in emergency and other high-risk organizations following exposure to primary or secondary trauma and to report on the effectiveness of the early intervention models in common use.

**Methods:**

A scoping review was conducted to examine early interventions for workers exposed to trauma, including emergency response, military, and humanitarian aid. Relevant data were extracted from the included studies and the outcomes were assessed using meta-ethnography.

**Results:**

Fifty studies of mixed quality met the inclusion criteria for this review. A synthesis of study outcomes found that early interventions help emergency responders to manage post-incident trauma when they are delivered in a manner that (a) respects distinct organizational culture, (b) is supported by organizations and senior management, and (c) harnesses existing social cohesion and peer support systems within teams.

**Conclusion:**

This review demonstrates that early interventions support emergency responders following exposure to trauma when these are tailored to the needs of the population, are supported by the host organization, and harness existing social cohesion and peer support processes within a team or unit. A number of recommendations for the delivery and evaluation of early interventions for psychological trauma in emergency response organizations were made.

## What Is Already Known About This Subject

• Some staff require support for mental health problems following organizational trauma exposure, yet their needs may be overlooked, and guidance has been inconsistent on appropriate models for early intervention.

• Early intervention for trauma may meet several needs for leaders and their teams, including valued support, social cohesion, reduction in harmful responses, reduced sick leave, and increased performance.

## What This Study Adds and Its Impact on Policy and Practice

• Identification of research which has examined the use of early interventions for trauma with staff in roles including emergency response, military, and humanitarian aid following exposure to primary or secondary trauma.
• In addition to collating information as to what intervention models are currently available, the synthesis of results allowed us to report on how early intervention models are delivered in organizational settings and provide guidance for organizations.

## Introduction

Traumatic events cause the most psychological damage when they occur without warning in situations both emotionally challenging and difficult to control (Paton and Violanti, 1996). In some organizations, including police (Regehr et al., 2019), ambulance (Petrie et al., 2017), fire and rescue (Lee et al., 2017), and health professionals (Somville et al., 2016), traumatic events are routine experiences for workers due to direct and indirect exposures (MacEachern et al., 2011; Skogstad et al., 2013). Following traumatic exposure, many workers experience upset and distress that may reduce their productivity, cause absence, and increase accidents and errors (McNally et al., 2003). In a group of traumatized emergency service workers, the perceived capability to perform at work was estimated to be 37% of their normal level of performance (Tehrani, 2020). For most, the psychological impact will reduce over the next few days and weeks. However, some may be affected and require support for later-onset mental health problems such as post-traumatic stress, anxiety, depression, and compassion fatigue (Huddlestone et al., 2006; Tehrani, 2016), yet their needs are often overlooked (Brandt et al., 1995).

It is important for trauma-exposed organizations to provide immediate support to their staff at the time of an incident. Brief crisis interventions are intended to ease emotional distress following exposure to trauma (Raphael and Wilson, 2000). In an organizational context, such as emergency services, early interventions are described as a group process involving a “meeting between the rescue worker and a caring individual (facilitator) able to help the person talk about his feelings and reactions to the critical incident” (Mitchell, 1983; p. 37). Early interventions are not designed to prevent or treat post-traumatic stress disorder (PTSD) (Ruck et al., 2013). However, the provision of an organizational early intervention following a traumatic incident can meet several needs for the leaders and their teams, including (a) facilitating mutual support for workers, (b) providing an opportunity to identify workers requiring additional clinical support, (c) increasing levels of social cohesion, (d) reducing harmful responses (e.g., alcohol abuse), (e) reducing levels of sick leave, and (f) improving workplace performance (Creamer et al., 2012; Regel and Dyregrov, 2012). Advocates of early interventions posit that the benefits are in its delivery soon after the traumatic exposure (usually between 2 and 10 days): the provision of psychosocial support, the opportunity to create a shared narrative of the trauma experienced, and the provision of stress education and management. In addition to mitigating distress, early interventions may also reduce the levels of sickness absence in trauma-exposed employees (McNally et al., 2003). The three most commonly used post-trauma interventions in organizations are critical incident stress debriefing (CISD; Mitchell, 1983), psychological debriefing (Dyregrov, 1989), and trauma risk management (TRiM; Jones et al., 2003), all of which are based on trauma-focused debriefing principles.

Previous reviews into the success of post-trauma interventions have shown mixed results. In 2005, the National Institute for Health and Care Excellence (NICE), a United Kingdom body which provides guidance and advice on improving health and social care, carried out a number of reviews of practice regarding the management of PTSD in adults and children (NICE, 2005, 2018a). While the NICE analysis found no evidence of any significant reduction in PTSD symptoms following psychological debriefing, it acknowledged that it was good practice to provide practical and social support and guidance to those affected by a traumatic incident. NICE examined several studies using models of debriefing involving single sessions of variable content and duration rather than a standardized protocol for group debriefing within an organization. One of these studies, undertaken on hospital patients who had suffered burns (Bisson et al., 1997), found an increase in trauma symptoms at 13 months post-injury. Based on these studies, the NICE development group concluded that brief, single-session interventions following a traumatic incident were not recommended (Hawker and Hawker, 2015).

NICE updated its guidelines on PTSD (NICE, 2018a) and accepted that the quality of evidence in developing the guidance for early interventions was low, which is reflected in the decision to not make any recommendations for early psychosocial interventions for adults (p. 154). It was recognized by NICE (2018b) in its response to stakeholders (p. 330) that its guidance was not designed to address the needs of emergency responder organizations in providing psychosocial interventions to trauma-exposed staff. The use of early trauma interventions in organizations and community settings for the purposes of social cohesion, education, personal well-being, and support is instead more appropriately located in occupational and public health bodies more knowledgeable in the evaluation of organizational interventions. As the NICE development group stated: “Occupational groups have campaigned to have the psychological impact of their work recognized and support services delivered as part of their conditions of employment. In addition, in military organizations, there exists a specific drive to early interventions—that of enabling traumatized combatants to return to frontline duties as soon as possible” (NICE, 2005, p. 81). Some organizations have subsequently chosen not to use any form of debriefing with their staff (Jones et al., 2003) despite the NICE guidance stating that its recommendations relate to the use of debriefing as a treatment rather than as a tool of community support or social cohesion. In this context, it is clear that further work is required to establish what should be considered best practice in terms of early post-trauma interventions for organizations (Hawker et al., 2011; Dyregrov and Regel, 2012).

The objectives of this review were to identify research which has evaluated the use of early interventions following exposure to primary or secondary trauma and to report on the personal effectiveness and organizational benefits of the commonly adopted early intervention models. The focus was on interventions taking place within the first month following a traumatic exposure (i.e., early interventions). The scope of this review was inclusively examining a range of intervention studies. The aim was to identify the elements that made early intervention models successful. The synthesis of study outcomes allowed for recommendations for the delivery of early interventions.

## Method

### Search Strategy

One literature search of the databases Embase, Global Health, Health Management Information Consortium, MEDLINE, and PsycINFO combined trauma terms, including “psychological trauma,” “burnout,” and “distress,” terms relating to early interventions, such as “debriefing,” “stress management,” and “post trauma,” and terms relating to emergency services and other occupational groups such as “rescue” and “police” (for a full list of search terms, see Supplementary Information 1). The journal(s) of Traumatic Stress, Emergency Medical Services, and Mass Emergencies and Disasters were hand-searched across all years. Conference proceedings were searched for relevant publications. The resulting citations were downloaded to EndNote version X8 (EndNote, Philadelphia, PA, United States). Titles, abstracts, and full texts were screened against the inclusion criteria by author MTR. The selections were reviewed by LG, DW and HC, with a discussion among the authors to resolve any uncertainty. The reference lists of the remaining articles were then hand-searched for additional relevant studies.

### Inclusion Criteria

The articles were eligible for inclusion if they:

• Were written in English,
• Included original (experimental) data, whether qualitative or quantitative,
• Examined an early intervention for trauma with members of any occupational service potentially exposed to trauma, whether the exposure is expected or unexpected,
• Examined the impact of an early intervention for trauma on mental health outcomes, social outcomes, and/or organizational outcomes, and
• Involved subjects who were exposed to trauma as a result of their employment.

### Data Analysis

Meta-ethnography (Noblit and Hare, 1988) was used for the analysis of the included studies. Meta-ethnography allows for a reciprocal translational analysis approach wherein the concepts can be “translated” from individual studies into one another, resulting in “lines of argument” (Britten et al., 2002). Primary themes (first-order constructs) and secondary themes and concepts (second-order constructs; interpretations by study authors) were identified. Synthesis involved determining relatedness by examining the primary and the secondary themes across studies and developing third-order constructs (reviewer interpretations; Atkins et al., 2008). This stage was performed by one of the authors (MR).

### Quality Appraisal

Downs and Black’s checklist for assessment of healthcare intervention methodology was used to appraise the risk of bias and the quality of the included studies (Downs and Black, 1998). This tool assesses quality in five areas—reporting, external validity, internal validity (bias), internal validity (selection bias), and power. Scored across 27 individual items, the studies with higher summed totals indicate comparatively higher quality to other included studies.

## Results

**FIGURE 1:**
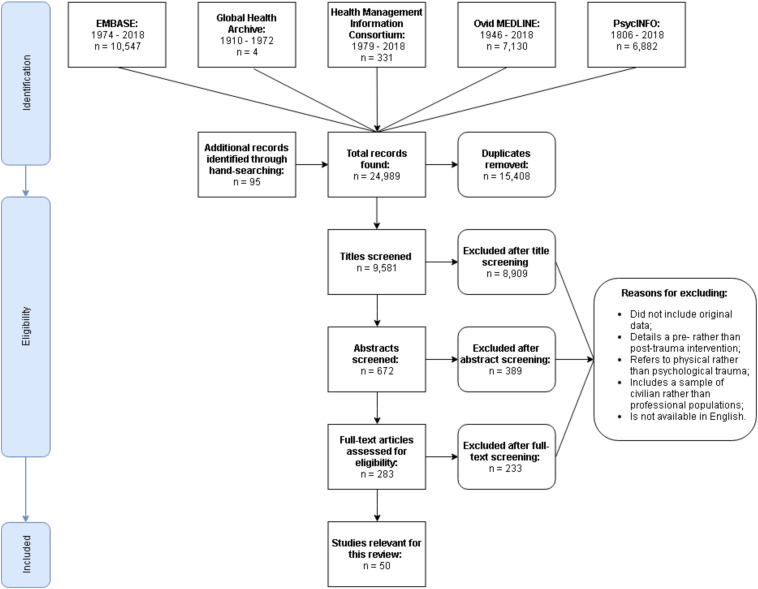
PRISMA flow diagram detailing the database search.

The initial search yielded 24,989 studies. Of these, 283 were relevant to the topic of early interventions for trauma and 50 were relevant for inclusion in this review (Figure 1).

### Study Characteristics

Seven (14%) studies contained qualitative data, 14 (27%) were longitudinal, and 10 (20%) were randomized controlled trials. The disasters described in the studies included natural disasters (*n* = 5), terrorist attacks (*n* = 4), peacekeeping in a conflict zone (*n* = 15), healthcare emergencies and patient fatalities (*n* = 3), shootings (*n* = 2), automotive/air/rail accidents (*n* = 5), public suicide (*n* = 2), and interviews with victims of child abuse (*n* = 1). In 10 studies, the incident varied between participants and three did not disclose specific details. The occupations included the military (*n* = 18), medical/health care (*n* = 9), police (*n* = 8), disaster responders (*n* = 6), fire fighters (*n* = 4) plus one study involving charity workers, researchers, prison officers, and retail and postal workers.

**FIGURE 2:**
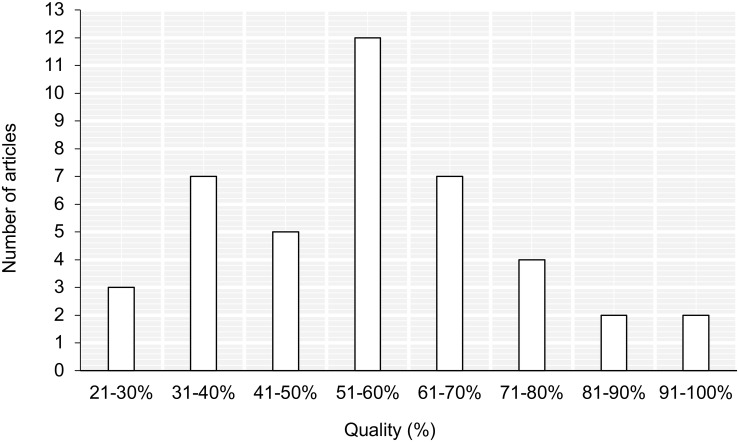
Quality appraisal scores for the included studies.

Overall, the study quality was mixed (Figure 2), tending to be strongest in reporting the methodology and the results (over 95% provided a clear description of measures and outcomes; 76% described the intervention in detail). The scores for internal validity were mixed: 52% of the interventions adhered to previously established protocols. In fewer than half, the authors adjusted for confounding variables (such as baseline trauma scores or prior exposure) and only a third randomized the participants to intervention groups (see Supplementary Information 2 for the summaries of all included studies).

Over half (66%) of the studies that fit the definition of an early intervention had a positive effect on PTSD symptom severity, work-related outcomes (absences), or self-reported quality of life. Two interventions (4%) had an adverse effect (Belton, 2017; Grundlingh et al., 2017). The remaining interventions revealed no significant difference between treatment and assessment-only controls.

The papers were evaluated individually for efficacy in supporting workers following a critical incident, and approximately half using CISD had a positive effect on some measures of PTSD symptom severity (Macnab et al., 1998; Leonard and Alison, 1999; Deahl et al., 2000; Mitchell et al., 2000; Adler et al., 2008; Hutton et al., 2010; Ruck et al., 2013; Grundlingh et al., 2017), whereas two found an adverse effect (Matthews, 1998; Harris et al., 2011). For example, Carlier and colleagues found no difference between intervention and control groups on symptom severity or organizational indexes of impact, such as sickness absence (Carlier et al., 2000). In 81% of CISD-based studies, the participants felt that the intervention was beneficial and helped them through recovery.

Two out of four studies assessing TRiM reported that peer group debriefings led to significant reductions in risk assessment scores and trauma-related sickness absences (Frappell-Cooke et al., 2010; Hunt et al., 2013). Two studies (Greenberg et al., 2010; Jones et al., 2017) found no difference between pre- and post-intervention trauma and anxiety.

Nineteen studies assessed the impact of non-specific “debriefing”—although the procedures and the focus of the intervention differed between studies. In 11 (58%), debriefing had positive gains for emergency responders, such as on emotions and meaning (Robinson and Mitchell, 1993; Kenardy et al., 1996; Chemtob et al., 1997; Shalev et al., 1998; Regehr and Hill, 2001; Tehrani et al., 2001; Halpern et al., 2009; Palgi et al., 2012; Wu et al., 2012; Firing et al., 2015; Gunasingam et al., 2015). Tehrani and colleagues described the group debriefing session delivered to the employees following a response to a rail accident (Tehrani et al., 2001). The researchers noted how the staff’s attitudes appeared to improve even during the debriefing, moving from regret at missed opportunities to appreciation of what they achieved through their response. Of the remaining debriefings, six (32%) had no effect on psychiatric morbidity (Deahl et al., 1994; Rick et al., 2006; Adler et al., 2009; Brandt et al., 2009; Blacklock, 2012; Shoval-Zuckerman et al., 2015) and two (11%) had an adverse effect on symptom severity (Carlier et al., 1998; Belton, 2017). Many of those taking part expressed how debriefing had been beneficial to them personally. Other early interventions identified included psychological first aid, which had little to no effect on PTSD symptom severity (Biggs et al., 2016).

### Meta-Ethnography

Five key concepts were identified: adherence, organizational context, governance, social support, and perceived benefits. These were linked together in a line of argument that accounts for how well an early intervention mitigates PTSD symptom severity in emergency responders following exposure to trauma (Table 1). The full meta-ethnography of the included studies is summarized in Supplementary Information 3.

**TABLE 1:** Synthesis, including concepts and second- and third-order interpretations.

Concepts	Second-order interpretations	Third-order interpretations
Adherence: (In)appropriate adoption/adaptation of intervention protocols Organizational context: Requirements to adjust models for specific organizations; target populations	(a) The interventions vary by their compliance with established protocols. Where a study departed from recommended methods, this was most often because of additional barriers that are specific to working with emergency response organizations. The nature of these challenges varies from logistical (e.g., work load), ethical, and legal (e.g., withholding treatment during randomized controlled trials) to cultural (e.g., stigma and fears of impact to career progression)	(b) The original authors note that the efficacy of an intervention relies upon proper adherence to tried and tested protocols. However, many practitioners highlight the necessity to adapt protocols to meet the needs of their targeted population, for example, accounting for the heavy workload of emergency responders. The studies also point to the importance of addressing distinctive organizational culture (e.g., perceived stigma) before an intervention can support posttraumatic recovery
Governance: Facilitated by the organization, included in standard protocols, and involves managers in the process	(c) There is an overlapping need to implement support programs at the organizational level, including formalizing treatment into primary care and involving managers or commanding officers, to broadcast a supportive workplace climate	(d) A mandated program aids post-incident recovery by reducing the stigma associated with help-seeking, presents a culture of support from peers and management, and delivers on organizations’ duty of care toward its workers
Social support: Peer advocacy; collective vs individual coping	(e) The studies show that group-level discussions have reliable positive effects for pre-existing teams and units	(f) Collective coping promotes recovery in a number of ways from practical (e.g., to construct a faithful account of the incident and for sharing coping strategies) to psychological (e.g., reconnecting workers to their communities and providing a sense of belonging)
Perceived benefits: The participants did or did not receive subjective (compared to objective) gains following the intervention	(g) Whether interventions significantly reduce symptom severity or not, the participants derive subjective value, appreciation, and satisfaction from debriefings	

#### Adherence

The studies can be separated into those adhering to previously outlined protocols and those that have been modified. Of the interventions that adhered to established protocol, fewer than half (43%) had a positive effect on symptom severity. Of those described to have been catered to the needs of emergency responders, 65% reported positive gains. The most common deviations were in the timing of delivery. For example, Blacklock describes the delivery of CISD to healthcare professionals following a suicide on hospital grounds (Blacklock, 2012). Rather than targeting two windows of opportunity for trauma management [as identified by the original authors; Mitchell (1983)], the researchers co-joined defusing (recommended for the first 24 h) and formal debriefing (recommended for the first 72 h) into a single session.

#### Organizational Context

The requirement to adjust the models stems from challenges specific to emergency response, varying from logistical to cultural. For example, modifying intervention models by reducing them into a single session helps to “capture the maximum amount of (nursing and medical) staff,” who might otherwise be forced to ignore or leave posttraumatic stress unattended (Blacklock, 2012, p. 4). The success of implementing effective support in military populations who are often transferred between units and separated from support networks depends on the flexibility and the duration of the model (Rudd et al., 2015).

The emergency response staff cited a culture of stigma in their organization as being a significant barrier to help-seeking and recovery. During CISD, healthcare professionals listed loss of professional integrity and impact on career prospects as preventing support-seeking after the traumatic loss of a patient (Hutton et al., 2010). The perspective of peers also plays a significant role on whether support is efficacious. For example, 17 police officers given the opportunity to discuss their experiences following trauma exposure expressed having been “mildly teased” by peers who were not involved (Young and Parr, 2004).

#### Governance

The included papers indicated governance to be an important factor in predicting the efficacy of early interventions. The study authors and the participants spoke about the benefits of implementing programs of support into a standard operating procedure. For example, the police officers appreciated receiving CISD because it came with a fully mandated program of care (Becker et al., 2009). The military officers likewise preferred debriefing to be classified as primary care rather than as a mental health appointment as it lessened the stigma surrounding help-seeking (Cigrang et al., 2017). If all personnel are required to attend a debriefing, it gives the impression that the employers are “benevolent enough to provide support” (Blacklock, 2012). Grundlingh and colleagues assessed the effectiveness of group debriefings delivered to 59 assistant researchers exposed to secondary trauma after interviewing victims of child abuse (Grundlingh et al., 2017). The results revealed that the debriefings were not any more effective in reducing distress over simply engaging in a leisurely activity, but the staff were less likely to report emotional distress when they perceived their organization to be supportive. This also affects organizational efficiency: the more employees feel positive about the support provided by their organization, the less time they spend off work (Rick et al., 2006).

The managers were found to be uniquely capable of creating either a safe learning climate for reflection (Firing et al., 2015) or a culture of criticism, blame, and stigma (Halpern et al., 2009). In many of the included studies, the workers highlighted the importance of having the support of their supervisors or departmental chiefs (Brandt et al., 1995). In two studies, the supervisors were considered an important source of support for reducing stress in police officers (Chongruksa et al., 2012) or for feeding workplace outcomes back to study evaluators (Chongruksa et al., 2015). In studies where a manager/commander was involved (either during referral, facilitating the intervention itself, or providing feedback) or where the organization presided over the early intervention process, 81% (21 out of 26) found that the intervention had positive effects on measures of symptom severity, quality of life, or workplace outcomes. In those studies where the organization did not directly govern the intervention, only 36% (nine out of 25) found the intervention to be beneficial for recovery. In summary, the success of post-trauma support appears to rely upon organizational acceptance from both colleagues and managers.

#### Social Support

Many intervention models (particularly CISD and TRiM) are designed to emphasize peer processes, reduce distress through collective recovery, and restore group cohesion and unit performance (Greenberg et al., 2010). Of studies delivering an early intervention in a group-based format, 74% (25 out of 34) found that peer support had facilitated recovery or had made for a better experience. For example, Armstrong et al. delivered group debriefings to American Red Cross workers following their response to a Los Angeles earthquake (Armstrong et al., 1998). During the intervention, the participants were invited to construct a group narrative of the event and to share coping strategies which the participants found helpful. In another study where team support was low, the employees exhibited higher levels of trauma-related stress (Frappell-Cooke et al., 2010).

An opportunity to discuss a critical incident with peers promotes posttraumatic recovery (Firing et al., 2015). From a practical perspective, group debriefing allows employees to construct a faithful account of the event, to fill in gaps in knowledge or memory, and to translate the experience into factual unemotional language. From a psychological perspective, collective recovery capitalizes on social cohesion within teams and units, reinforces that reactions are normal and shared by others, and helps reintegrate the employees back into the workforce (McNally et al., 2003; Greenberg et al., 2010).

#### Perceived Benefits

The participants evaluated the interventions to be subjectively useful even when the symptom severity scores suggested the contrary. For example, Matthews (1998) evaluated CISD delivered to psychiatric workers a week after being assaulted by a client (Matthews, 1998). The debriefed participants reported more work-related stress and PTSD symptoms compared to those who were merely assessed. However, almost 60% of the debriefed participants reported that it had helped them cope and reduce their feelings of stress. A large sample of military personnel positively evaluated their experience with debriefing, which correlated negatively with their scores of PTSD symptom severity (Belton, 2017). Of the debriefings that had no substantive effect on symptom severity, 78% (21 out of 27) were subjectively evaluated to be helpful.

The perceived benefits of early interventions include appreciation of the therapeutic climate that the debriefing created wherein the symptoms are openly discussed (Blacklock, 2012), how sharing the experience with others helps to integrate inner experiences with the outside world (Brandt et al., 1995), putting impressions into words to help in the recovery (Firing et al., 2015), and acknowledgment that the incident was “critical,” thus serving to normalize reactions (Halpern et al., 2009).

## Discussion

The aim of this review was to evaluate interventions for the early management of posttraumatic stress in emergency response organizations and to assess organizational benefits. This was to allow for the identification of the key components of early interventions and to make recommendations for their delivery to trauma-exposed staff in the workplace.

The included studies differed by the intervention and the measures used for assessment. The participants were all emergency response staff and others were employed in delivering support in the context of that role. Primarily, the interventions described group debriefing; however, a small number described support more appropriately categorized as trauma therapy or prevention. Trauma therapy differs to debriefing in terms of the timing of the intervention, the role and the experience of the facilitator, and the intended outcomes. Our focus was to evaluate interventions taking place within the first month following a traumatic exposure (i.e., early interventions).

Most early interventions were based on psychological debriefing which seeks “to prevent the development of adverse reactions” before they arise (Dyregrov, 1989, p. 25). Some interventions were described as one-on-one defusing with a manager or supervisor (within the first 24 h), but the majority involved debriefing within a group setting, focusing on narrative construction and social cohesion to support post-incident recovery. In recent decades, reports demonstrating that debriefing has either no effect (Roberts et al., 2009) or negative effects on PTSD symptom severity have been published (Rose et al., 2002)—serving only to aggravate post-incident distress. In our review, most early interventions led to reduced symptom severity. In the 12 studies where the severity scores did not change, half were still evaluated to be helpful for the participants.

There are several limitations to this study; as identified, the quality of some of the studies was low, which may have affected the findings. It is hoped that future studies will be undertaken with more robust experimental designs. There is a discrepancy between symptom scores and subjective evaluation, which may indicate that the positive effects of debriefing may be short, lasting briefly while the participants complete the evaluation forms but not long enough to influence a follow-up assessment. The participants may evaluate the experience of debriefing as different to and separate from posttraumatic recovery. For example, Adler et al. reported that CISD is well liked and well received by the participants but that the participants did not necessarily find it effective in reducing the symptoms (Adler et al., 2008). It is possible that debriefing may impart benefits not captured by existing outcome measures (Deahl et al., 2000). For example, fire service personnel receiving CISD following a motor vehicle accident found no significant effects of CISD on the Impact of Events Scale (relative to psycho-education or assessment-only controls), but those who had been debriefed were significantly less likely to consume alcohol as a means of coping and significantly more likely to report better quality of life (Tuckey and Scott, 2014). To uncover the benefits of early interventions, additional outcome measures may be needed.

The issue of measurement also highlights the issue of intervention design and scope (Dyregrov, 1989, 1998). Early interventions primarily act as a means to screen and manage immediate post-incident distress and to alleviate stress reactions triggered by critical events (Mitchell, 1983). It may not be reasonable to expect the debriefing interventions to impact measures used in PTSD diagnosis (Weiss and Marmar, 1996; Orsillo, 2001).

While some meta-analyses have shown that debriefing does not facilitate recovery (Rose et al., 2002; van Emmerik et al., 2002), other studies have shown it to have adverse effects. Bisson et al. (1997) found that, at 13 months, the PTSD rates were significantly higher in those debriefed compared to controls. In our review, only two studies showed that group debriefing had an adverse effect on symptom severity, but the protocol in these studies was ambiguously defined. For example, Belton (2017) reported that soldiers returning from deployment exhibited increased posttraumatic stress following mandatory debriefings. Importantly, the authors described the debriefing as generalized “rather than [using] any one specific [intervention] model” (Belton, 2017, p. 52).

This highlights one emerging theme synthesized from this review: adherence—many interventions departed from an established protocol. Dyregrov (1989, 1998) stipulates that debriefing should be delivered in a group setting and instigated within a brief period after the event, led by a trained and experienced facilitator. Studies mostly adhered to these requirements. However, in some cases, the protocol was modified (e.g., was delivered one on one rather than to a group). The subsequent intervention had no effect on symptom severity (Carlier et al., 2000; Harris et al., 2011). Dyregrov also argues that studies cited in the “debunking” of psychological debriefing suffered methodological issues (Dyregrov, 1998). This refers to issues of timing, length of session, and participants self-selecting to treatment conditions. While debriefings were sometimes delayed past the recommended window of opportunity, modifications were often made as a requirement for meeting discrete organizational needs. For example, Mitchell and colleagues found that police constabularies delivered debriefings within the recommended 48–72 h following an incident. However, almost a third had to delay support to account for officer availability (Mitchell et al., 2000). Cigrang et al. likewise delivered shorter-than-recommended sessions to overcome logistical issues specific to the military (Cigrang et al., 2017).

It is also often the case that emergency response organizations have a culture which devalues emotional vulnerability (Halpern et al., 2009). Many studies referred to workplace cultures that emphasize tough-mindedness and stigmatize ill mental health (Deahl et al., 1994; Kenardy et al., 1996; Young and Parr, 2004; Becker et al., 2009; Halpern et al., 2009; Frappell-Cooke et al., 2010; Chongruksa et al., 2015; Cigrang et al., 2017; Jones et al., 2017). This often results in reluctance to seek support. For example, police officers and staff are often nervous that asking for psychosocial help could impact on career progression (Hesketh and Tehrani, 2018). To overcome workplace barriers, the support staff must consider the wider context in which a critical incident is experienced before imposing an intervention model. In this review, greater successes were achieved when the practitioners modified an established protocol to address organizational barriers (e.g., Blacklock, 2012). Thus, while it is recognized that interventions should stick to validated models, there is also a need to appreciate organizational culture and understand that one size will not fit all when it comes to early interventions. Dyregrov stresses that indeed flexibility is important when it comes to good crisis intervention (Dyregrov, 2003).

We found that the TRiM and the CISD models were quantifiably more effective in facilitating recovery following trauma exposure than non-specific debriefing and brief early interventions such as psychological first aid. The relatively higher success rates of TRiM may be, in part, due to the formalized nature of the intervention, the perceived investment from commanders/managers, or the emphasis TRiM places on reducing stigma surrounding help-seeking (Watson and Andrews, 2017). It also includes delivery of interventions by a peer from within the same unit, circumventing logistical barriers like security vetting, and making it easier for peer supporters to identify unfolding issues.

Organizational support serves to reassure workers and facilitate recovery (Frappell-Cooke et al., 2010). This is reflected in the theme of governance. Governance refers to an overlapping need for organizations to formally implement early interventions into occupational health provision. Internal peer and professional support meets several needs for teams: it creates room for reflection and a supportive learning climate (Firing et al., 2015), it assists in coordinating and referrals of staff to formal assessment (Rudd et al., 2015), it delivers on employer expectations in provision of a safe environment (Ruck et al., 2013), and it serves to increase worker performance (Creamer et al., 2012). Line managers play a particularly important role in the governance of an early intervention. Mitchell and Stevenson found that supportive supervisors with a positive management approach reduced the likelihood of psychological problems arising (Mitchell et al., 2000).

On the other hand, the staff may be suspicious of the occupational health and senior management’s intentions rather than thankful for their support. For example, a qualitative study by Drury et al. (2013) reported disagreement among first responders of the extent to which line managers (and more broadly, organizations) provide adequate psychosocial support. [Halpern et al. (2009), p. 141] found that when supervisors were seen as being unsupportive of their employees’ well-being, they were described in “angry, resentful, and disappointed tones” by emergency medical staff, leading them to be distrusting of management having their best interests in mind. Macnab and colleagues likewise found distrust between medical staff unions and hospital senior management (Macnab et al., 2004). The importance of governance in this case may be more relevant to organizations with more clearly defined hierarchical structures, such as the police, the fire fighters, and the military. For example, studies illustrate how group debriefings are consistent with military tradition of after-action reviews, often delivered by unit commanders (Shalev et al., 1998; Deahl et al., 2000; Shoval-Zuckerman et al., 2015). Early interventions are indeed acceptable among military personnel when “fully supported by military commanders” (Jones et al., 2017, p. 237).

Our findings, together with prior reports, suggest that managers and commanders need to be involved and trained to spot and respond to mental health issues in the emergency response staff (Hesketh and Cooper, 2017). To create an open and safe environment, the senior management also needs to implement support programs at an organizational level as well as provide comprehensive training in advance of potentially traumatic experiences (Castro et al., 2006).

We found consensus among constituent studies that emergency responders benefitted from the opportunity to discuss their experiences and reactions to a traumatic incident with their peers. This was further supported by our meta-ethnography which identified the importance of social support in recovering from a traumatic exposure. Being debriefed with peers promotes recovery by recognizing their experiences in a familiar setting (Tehrani and Hesketh, 2018), allowing them to put their experiences into words (Firing et al., 2015), filling in gaps of knowledge, achieving greater understanding of the event, and curtailing feelings of detachment or loneliness (Olff, 2012). These interventions are highly valued in building social cohesion and support (Dyregrov, 2003). Although the use of debriefing has been challenged as a treatment for PTSD (Bisson et al., 2007), our review suggests that early interventions can support emergency responders when they cater to the specific needs of the population, are governed by the host organization and supported by management, and harness existing social cohesion and peer processes within a team or unit.

The outcomes of this review indicate that early interventions can be effective in organizations if they are conducted appropriately and according to evidence-based criteria. The effectiveness of providing early intervention support will not be optimal unless they are fully integrated into working practice.

We identified the following factors as important in the delivery and the evaluation of early interventions for psychological trauma in emergency response organizations:

• The importance of adhering to key components of the chosen intervention model.
• While some variations were beneficial in that they addressed cultural, organizational, and resourcing issues, sometimes these variations can be detrimental. Based on the included studies, it is not possible to determine whether varying from protocols significantly influenced well-being or intervention efficacy.
• Providing support for employees requires understanding of organizational cultures. Intervention success is increased when the practitioners cater to specific needs and work to overcome logistical (e.g., workload) and cultural (e.g., stigma) barriers.
• The most significant benefits from early interventions occur when part of a program of organizational support. Managers were particularly important in the referral and the assessment of work-related outcomes as they assign organizational resources and create a supportive and accepting workplace environment.
• Within the initial window of opportunity (before formal therapy), peer group processes are important in the management of post-incident stress, buffering significant issues that may appear down the line. In this review, recovery was more likely (i.e., less likely to need formal occupational health intervention or referral to clinical treatment) when emergency responders supported one another.
• Employees derive subjective satisfaction and appreciate the opportunity to discuss their experiences. The results also indicate that the objective measures of PTSD do not fully capture the potential positive outcomes from an early intervention.

Additional assessments are needed of early interventions that incorporate outcomes characterizing benefits aligned with social well-being. This might include measures of engagement in potentially harmful behaviors, such as alcohol reliance, as well as organizational benefits, including length of absence from work. The next steps should also include the development of tools and guidance appropriate for the provision of context-specific early intervention procedures such as within policing. This may take the form of identifying individual elements of recognized models and the evidence for its effectiveness to create a template for training within services.

## Author Contributions

NT, IH, and RA conceived the study and, with DW, HC, LG, and MR, designed the search strategy, advised on data collection, and supported the analysis of the data. MR screened the search results, performed data extraction and analysis, and interpreted the data. MR and LG prepared the manuscript. All the authors reviewed, commented on, and approved the manuscript.

## Conflict of Interest

The authors declare that the research was conducted in the absence of any commercial or financial relationships that could be construed as a potential conflict of interest.
