# Supplementary material for: Early Post-trauma Interventions in Organizations: A Scoping Review
Source: Front Psychol. 2020 Jun 25;11:1176. doi: 10.3389/fpsyg.2020.01176 (PMC7330139; doi:10.3389/fpsyg.2020.01176)
Supplement: Supplementary file 1 [file Table_1.docx]

**Supplementary Material 1 –** Search terms

1 – 20: Early interventions

22 – 39: Emergency response and occupational relevance

41 – 60: Psychological trauma

| 1 | (Early adj3 intervention*).tw. |
| --- | --- |
| 2 | Debrief*.tw. |
| 3 | (Victim adj5 (witness* or support)).tw. |
| 4 | (Training adj3 (trauma* or stressor* or support* or emergenc*)).tw. |
| 5 | (Social adj3 (support or well?being or intervention)).tw. |
| 6 | (Spiritual adj3 (support or well?being or intervention)).tw. |
| 7 | CBT.tw. |
| 8 | Psychological first aid.tw. |
| 9 | (Focus* adj3 support).tw. |
| 10 | Critical Incident Stress Debrief*.tw. |
| 11 | Critical Incident Stress management.tw. |
| 12 | Trauma Risk Management.tw. |
| 13 | support post trauma.tw. |
| 14 | (Individual* adj5 (debrief* or support* or intervention)).tw. |
| 15 | (Communit* adj5 (debrief* or support* or intervention)).tw. |
| 16 | (Group adj5 (debrief* or support* or intervention)).tw. |
| 17 | (Psycholog* adj3 model*).tw. |
| 18 | exp Social Support/ |
| 19 | exp Peer Group/ |
| 20 | exp Crisis Intervention/ |
| 21 | or/1-20 |
| 22 | Organi?ation*.tw. |
| 23 | Staff.tw. |
| 24 | Emergenc*.tw. |
| 25 | Rescue.tw. |
| 26 | Officer*.tw. |
| 27 | Police.tw. |
| 28 | Fire.tw. |
| 29 | Ambulance.tw. |
| 30 | Military.tw. |
| 31 | Soldier*.tw. |
| 32 | Soldier Peer Mentoring.tw. |
| 33 | Humanitarian.tw. |
| 34 | Post office.tw. |
| 35 | Royal mail.tw. |
| 36 | Transport.tw. |
| 37 | exp Occupational Disease/ |
| 38 | exp Law Enforcement/ |
| 39 | exp Rescue Work/ |
| 40 | or/22-39 |
| 41 | exp mental health/ |
| 42 | (Post adj3 trauma*).tw. |
| 43 | exp PTSD/ |
| 44 | exp Anxiety/ |
| 45 | (compassion adj3 fatigue).tw. |
| 46 | Shock.tw. |
| 47 | Fear.tw. |
| 48 | Emotion*.tw. |
| 49 | Burnout.tw. |
| 50 | Sleep.tw. |
| 51 | Violen*.tw. |
| 52 | (Abus* adj3 (substance* or drug*)).tw. |
| 53 | (trauma adj (primary or secondary)).tw. |
| 54 | Well?being.tw. |
| 55 | exp Trauma/ |
| 56 | exp psychological trauma/ or stress disorders, post-traumatic/ or stress disorders, traumatic, acute/ |
| 57 | (adverse adj3 effects).tw. |
| 58 | (Post adj3 trauma).tw. |
| 59 | (Expos* adj5 (stress* or trauma*)).tw. |
| 60 | (professional adj3 burn?out).tw. |
| 61 | or/41-60 |
| 62 | 21 and 40 and 61 |
| 63 | limit 62 to english language |
