# Supplementary material for: Early Post-trauma Interventions in Organizations: A Scoping Review
Source: Front Psychol. 2020 Jun 25;11:1176. doi: 10.3389/fpsyg.2020.01176 (PMC7330139; doi:10.3389/fpsyg.2020.01176)
Supplement: Supplementary file 2 [file Table_2.DOCX]

**Supplementary Material 2**

Table 1. Summary of included papers.

| **Study citation** | | **Design** | **Incident described** | **Sample** | |  | | **Occupation** | | **Intervention** | | **Outcome assessed** | **Results** | | | **Delivery details** |
| --- | --- | --- | --- | --- | --- | --- | --- | --- | --- | --- | --- | --- | --- | --- | --- | --- |
|  | |  |  | **N** | **f/m** | **Age**  **(m, sd)** | |  | |  | |  |  | | |  |
| Adler et al. (2008) | | 1. 3 groups (CISD vs. SMC vs. SO);  2. Randomly allocated;  3. Pre- and post-data. | Peacekeeping in a conflict zone | 952 | 28 / 924 | undisclosed | | Military personnel | | CISD; SMC; SO | | PCL; HR; POS; CES-D; CTS | At high levels of exposure, CISD was associated with decreases in PCL compared to SMC, d =.12; SMC was associated with slight increases, compared to SO, d = .11; SO and CISD did not differ at any level of exposure. | | | 1. Multi-session;  2. Groups;  3. Day-trained instructors;  4. "not tied to a specific discrete event". |
| Adler et al. (2009) | | 1. 3 groups (psychoeducation vs. debriefing vs. small Battlemind training vs. large Battlemind training);  2. Randomly allocated;  3. Pre- and post-data. | Peacekeeping in a conflict zone | 2,297 | 96 / 2191 | undisclosed | | Military personnel | | debriefing | | SUDS; PCL; PHQ | At follow-up, Battlemind training in large groups led to lower PHQ scores as compared to psychoeducation, p = .05; there was no significant difference between debriefing and small group training on any outcome, ps < .05; there was no difference in group size of Battlemind training, p < .05. | | | 1. Multi-session;  2. Groups;  3. Research team;  4. 72-hours after exposure. |
| Armstrong et al. (1998) | | 1. 2 groups (individual vs. group);  2. No randomisation;  3. Only post-data. | Earthquake | 95 | 53 / 42 | 50, 13.7 | | American Red Cross workers | | MSD | | Self-reported evaluations | There were no significant differences between individual compared to group debriefing, p <.05; but allowing participants to have fuller discussions of their reactions and being able to share coping strategies with one another provided a better debriefing experience, p < .0001. | | | 1. Single session;  2. Individual and groups;  3. Mental health professionals;  4. 3-weeks after exposure. |
| Beaumont et al. (2016) | | 1. 2 groups (TF-CBT or TF-CBT + CFT);  2. Randomly allocated;  3. Pre- and post-data. | undisclosed | 17 | 5/12 | 42.25 | | Fire Service Personnel | | TF-CFT; CFT | | HADS; IES; SCS | TF-CBT + CFT was more effective than TF-CBT alone at increasing self-compassion, p=.05; TF-CBT + CFT did not significantly differ from TF-CFT alone on HAS or IES, but trended in that direction. | | | 1. Multi-session;  2. Groups;  3. Mental health professionals;  4. Undisclosed. |
| Becker et al. (2009) | | 1. 6 groups;  2. Self-selection;  3. Post-data only. | Description of a shooting | 379 | 110 / 269 | 32, 10.36 | | Police officers | | CISD; Exposure; CPT; EMDR; BEP; pharmacology | | CS; PDS | Those meeting the PTSD criteria rated either exposure or CPT as their first or second choice for treatment; psychopharmacology was the least preferred choice, followed by EMDR and psychodynamic. | | | n /a |
| Belton (2017) | | 1. 2 groups (debriefing vs. assessment only);  2. No randomisation;  3. Post-data only. | Peacekeeping in a conflict zone | 2,297 | 95 / 2,181 | undisclosed | | Military personnel | | debriefing | | PTSS; CES-D; DAS; NTDS. | Those who participated in debriefing had higher scores for PTSS (p<.01), CES-D (p<.05), and DAS (p<.001) than those who did not. There was no difference in NTDS scores. | | | 1. Multi-session;  2. Groups;  3. Mental health professionals;  4. 1-week after exposure. |
| Biggs et al. (2016) | | 1. 2 groups (PFA vs. assessment only);  2. Randomly allocated;  3. Pre- and post-data. | Peacekeeping in a conflict zone | 126 | 41 / 85 | 28.1, 7.2 | | Military personnel | | PFA | | PHQ; WHOQOL; CES; self-reported evaluations | The PFA intervention showed no overall effects on PCL, CES, or QOL (ps>.05). In males, PTSD symptoms increased after baseline, while decreasing in the comparison group, p <.05. | | | 1. Multi-session;  2. Groups;  3. Mental health professionals;  4. 2-7 months after exposure. |
| Blacklock (2012) | | 1. 1 group (no control group);  2. No randomisation;  3. Post-data only. | Public suicide | 43 | undisclosed | undisclosed | | Healthcare professionals | | Adapted CISM; combined defusing and debriefing | | IES | Following initial CISM session, all staff reported no further symptoms of intrusive thoughts or avoidant behaviours; 6 weeks later, 6 of 13 responders reported being sometimes affected by the incident and 7 said they were often affected. | | | 1. Single-session;  2. Groups;  3. Mental health professionals;  4. 7-hours after exposure. |
| Brandt et al. (2009) | | 1. 1 group (no control group);  2. No randomisation;  3. Post-data only. | Air accident | 252 | 92 / 160 | 30 | | Rescue workers | | debriefing | | Self-reported evaluations | Workers reported that sharing experiences in a debriefing group helped them "move from distancing to integrating their inner experience with the outside world". | | | 1. Single-session;  2. Groups;  3. Research team;  4. 1-week after exposure. |
| Carlier et al. (1998) | | 1. 2 groups (debriefed vs. assessment only);  2. No randomisation;  3. Post-data only. | Air accident | 105 | 10 / 95 | 37, 6.3 | | Police officers | | debriefing | | SI-PTSD | At 8 months, there was no difference in PTSD diagnosis (symptom or cluster level); At 18 months, officers who had been debriefed were significantly more likely to exhibit hyperarousal symptoms, p < .05. | | | 1. Multi-session;  2. Individual;  3. Mental health professionals and peers;  4. "as soon as possible". |
| Carlier et al. (2000) | | 1. 3 groups (CISD vs. assessment only vs. external control group);  2. No randomisation;  3. Pre- and post-data. | Varied in severity (from confrontation to child murder) | 243 | 40 / 173 | 31, 6.2 | | Police officers | | CISD | | SRS-PTSD; IES; PDEQ; ADIS | At post-test, there were no significant differences on any measure; no significant differences in sick leave or resumption; debriefed officers expressed greater satisfaction but there was no relationship between that and PTSD symptoms. | | | 1. Multi-session;  2. Individual;  3. Peers;  4. 24-hours after exposure. |
| Chemtob et al. (1997) | | 1. 2 groups (staggered treatment);  2. Counter-balanced;  3. Pre- and post-data. | Natural disaster | 43 | 34 / 9 | 41.9, 10.4 | | Disaster relief workers | | debriefing | | IES | IES scores decreased over time in group 1 and, later, in group 2; following debriefing, scores on avoidance and intrusion reduced significantly , p <.001. | | | 1. Single session;  2. Group;  3. Research team;  4. 6-months after exposure. |
| Chongruksa et al. (2012) | | 1. 2 groups (eclectic group counselling vs.psychoeducation only);  2. Random allocation;  3. Pre- and post-data. | Terror attack | 42 | undisclosed | 35.64, 9.99 | | Police officers | | CBT; religious; art therapy; reality therapy | | BDI; GHQ; SCL | Scores on BDI, GHQ, and SCL significantly reduced over time following post-eclectic group counselling, ps < .05, whereas psychoeducation alone had no effect. | | | 1. Multi-session;  2. Group;  3. Research team;  4. Undisclosed. |
| Chongruksa et al. (2015) | | 1. 2 groups (eclectic group counselling vs.psycoeducation only);  2. Random allocation;  3. Pre- and post-data. | Peacekeeping in a conflict zone | 44 | undisclosed | 30.34, 5.23 | | Military personnel | | CBT; religious; art therapy; reality therapy | | RS; TMHI; GHQ | Scores on RS, TMHI, and GHQ significantly reduced over time following post-eclectic group counselling, ps < .05, whereas psychoeducation alone had no effect. | | | 1. Multi-session;  2. Group;  3. Research team;  4. Undisclosed. |
| Cigrang et al. (2005) | | 1. 3 cases (no control group);  2. No randomisation;  3. Pre- and post-data. | Peacekeeping in a conflict zone | 3 | 0 / 3 | 23.33 | | Military personnel | | exposure therapy | | PCL | Each of the 3 soldiers showed reductions in PCL by an average of 56%, falling within sub-clinical ranges for PTSD; and by the end of the course, each reported feeling that they did not require any further treatment. | | | 1. Multi-session;  2. Individual;  3. Mental health professional;  4. 24-hours after exposure. |
| Cigrang et al. (2017) | | 1. 2 groups (staggered treatment);  2. Random allocation;  3. Pre- and post-data. | Peacekeeping in a conflict zone | 67 | 17 / 50 | 39.95, 8.65 | | Military personnel | | TF-CBT | | PCL; PHQ; BHM; PSS | Patients in the initial treatment group improved significantly on all five outcome measures, p< .05, with a clinically meaningful reduction (10 points or greater) in symptom severity on the PCL, compared to those in the delayed treatment group. | | | 1. Multi-session;  2. Individual;  3. Mental health professional;  4. Undisclosed. |
| Deahl et al. (1994) | | 1. 2 groups (debriefing vs. assessment only);  2. No randomisation;  3. Post-data only. | Peacekeeping in a conflict zone | 62 | undisclosed | 28 | | Military personnel | | Group debriefing | | GHQ; IES; self-reported evaluations | There was no difference in scores between those debriefed and those simply assessed on the IES, p > .20, or the GHQ, p > .20; half of the debriefed participants evaluated the debriefing to be 'useful'. | | | 1. Single-session;  2. Group;  3. Trained support workers;  4. "as soon as possible". |
| Deahl et al. (2000) | | 1. 2 groups (CISD vs. assessment only);  2. Random allocation;  3. Post-data only. | Varied in severity (from artillery shelling to body handling duties) | 106 | undisclosed | 24 | | Military personnel | | CISD | | HADS; IES; SCL; CAPS | Over time, HADS scores were significantly reduced in the debriefed group compared to the non-debriefed group, p = .008; however, following debriefing there was no change in IES or CAPS, and elevation in SCL scores. | | | 1. Single-session;  2. Group;  3. Trained instructors;  4. "immediately" after exposure. |
| Dickstein et al. (2013) | | 1. 2 groups (full vs. subthreshold PTSD);  2. No randomisation;  3. Pre- and post-data. | Peacekeeping in a conflict zone | 534 | 57 / 477 | 45.91 | | Military personnel | | CPT | | CAPS; PCL; BDI | There was a significant reduction in PCL scores over time in both full and subthreshold PTSD participants, p <.001, with an average decrease of 1.52 points at each time point; this indicates that CPT may be effective as an early post incident treatment of PTSD | | | 1. Multi-session;  2. Individual;  3. Mental health professionals;  4. Undisclosed. |
| Difede et al. (2007) | | 1. 2 groups (CBT vs. TAU);  2. Random allocation;  3. Pre- and post-data. | Terror attack | 22 | undisclosed | 45.77, 7.72 | | Disaster relief workers | | CBT-exposure | | CAPS; PCL; BDI; SAS; MAST | Following CBT treatment, scores significantly declined on the CAPS, p =.019, and PCL, p = .020 compared to TAU (at least a 10 point reduction); no effects observed on BDI or SCL or MAST. | | | 1. Multi-session;  2. Individual;  3. Mental health professional;  4. 8-36 months after exposure. |
| Drury et al. (2013) | | 1. 6 focus groups;  2. Delphi study. | Healthcare emergency | 101 | 34 / 67 | undisclosed | | First responders | | Psychoeducation | | Self-reported evaluations | Participants agreed on stress emerging from responding to serious injuries; identified need for peer-support training to recognise and navigate stress following response to incidents; support from line managers (trauma informed or clinically trained). | | | n /a |
| Firing et al. (2015) | | 1. In depth interviews;  2. Analysed using Interpretive Phenomenological Analyses. | Terror attack | 6 | undisclosed | undisclosed | | Search and rescue crew | | Holistic debriefing | | Self-reported evaluations | Participants commented on the importance of being able to normalise their feelings; to develop interpersonal relationships with their peers; to engage with their emotions on a deeper level; to comprehend the reasons for their individual reactions; to construct meaning. | | | 1. Multi-session;  2. Individual;  3. Research team;  4. 24-hours after exposure. |
| Frappell-Cooke et al. (2010) | | 1. 2 groups (experienced with TRiM vs. first use of TRiM);  2. No randomisation;  3. Pre- and post-data. | Peacekeeping in a conflict zone | 422 | undisclosed | undisclosed | | Military personnel | | TRiM | | GHQ; PCL | Those with experience of TRiM had lower GHQ scores than those using it for the first time, p < .01; both groups had higher GHQ scores before and during deployment, compared to post-deployment; Greater access to sources of social support was associated with lower GHQ and PCL scores, ps < .05. | | | 1. Multi-session;  2. Group;  3. Peers;  4. 72-hours after exposure. |
| Greenberg et al. (2010) | | 1. 2 groups (TRiM vs. TAU);  2. Random allocation;  3. Pre- and post-data. | Varied in severity (significant injury to natural disasters) | 1551 | 159 / 1392 | undisclosed | | Military personnel | | TRiM | | GHQ; PCL; perceived stigma | There was no difference between TRiM and TAU in terms of pre- and post-GHQ or PCL scores, ps >.05; there was also no effect of the intervention on internal or external stigma, ps >.05. | | | 1. Multi-session;  2. Group;  3. Peers;  4. 48-hours after exposure. |
| Grundlingh et al. (2017) | | 1. 2 groups (CISD vs. leisure);  2. Random allocation;  3. Pre- and post-data. | Interviews with victims of child abuse | 53 | 34 / 19 | 29.75 , 4.48 | | Violence researchers | | CISD | | SRQ; VTS; IES; STS | In both groups, SRQ remained unchanged over time; the CISD group (vs. control) had significantly higher IES and STS scores, p = .002; staff were less likely to report emotional distress when perceiving organisational support, p = .002. | | | 1. Multi-session;  2. Group;  3. Mental health professional;  4. Undisclosed. |
| Gunasingam et al. (2015) | | 1. 2 groups (debriefing vs. assessment only);  2. Random allocation;  3. Pre- and post-data. | undisclosed | 31 | 15 / 16 | undisclosed | | Junior doctors | | debriefing | | MBI; self-reported evaluations | MBI scores reduced post debriefing, whereas increased in the control group; the difference between groups was however not significant p = .83; in evaluations, 60% of participants suggested that they would recommend debriefing to peers and 90% found it to be a source of emotional and social support. | | | 1. Multi-session;  2. Group;  3. Mental health professional;  4. 1-hour after exposure. |
| Halpern et al. (2009) | | 1. focus groups and one-to-one interviews. | undisclosed | 100 | 33 / 67 | 39 | | Emergency Medical Technicians | | debriefing | | Self-reported evaluations | Participants highlighted the importance of: supervisor support and timeout within 24 h of the critical incident; perceived fear and stigma of appearing weak. Majority of participant wanted more education for themselves, their families and colleagues to recognise signs of critical incident stress. | | | n /a |
| Harris et al. (2011) | | 1. 2 groups (CISD vs. assessment only);  2. Random allocation;  3. Pre- and post-data. | undisclosed | 660 | 17 / 642 | 34.5 | | Fire Service Personnel | | CISD | | PSSS; WAS; WOCQ; IES; | There was no significant differences between the two groups on PSSS, WAS, WOCQ, and IES scores; there was, however, an inverse relationship between negative affectivity and CISD, p <.01; and a positive relationship between WAS scores and CISD, p <.05. | | | 1. Single-session;  2. Individual;  3. Research team;  4. 6-months after exposure. |
| Hunt et al. (2013) | | 1. 4 groups (TRiM, TRiM & 1:1, 1:1, assessment only);  2. No randomisation;  3. Post-data only. | Murder-suicide | 640 | 203 / 434 | 44.5 | | Police officers | | TRiM | | sickness absence | Risk assessment scores significantly reduced over time in the treated compared to untreated, group, p <.001; treatment (TRiM or TRiM + 1:1) was associated with a reduction in sickness absence, p <.001. | | | 1. Multi-session;  2. Group;  3. Manager;  4. 24-hours after exposure. |
| Hutton et al. (2010) | | 1. 2 groups (CISD vs. assessment only);  2. No randomisation;  3. Pre- and post-data. | Unexpected death of patient | 184 | undisclosed | undisclosed | | Healthcare professionals | | CISD | | Self-reported evaluations | Debriefed staff felt they managed their grief better than non-debriefed staff, p = .003; they also scored higher in their ability to maintain their professional integrity, compared to non-debriefed staff, p =.005; the vast majority of participants found the sessions helpful, informative, and helpful. | | | 1. Single-session;  2. Group;  3. Trained instructor;  4. 1-week after exposure. |
| Jones et al. (2017) | | 1. 3 groups (TRiM vs. non-exposed non-TRiM vs. exposed non-TRiM);  2. Random allocation;  3. Pre- and post-data. | Varied in severity (from artillery shelling to body handling duties) | 638 | 4 / 558 | undisclosed | | Military personnel | | TRiM | | GAD; PHQ; PCL; AUDIT; self-reported barriers to care | At follow-up, there was no difference between TRiM and non-TRiM groups on any mental health outcomes, ps > .05; TRiM recipients were significantly more likely to seek professional help, p < .05; TRiM recipients were more likely to report stigma than non-exposed, non-TRiM participants, p < .01. | | | 1. Multi-session;  2. Individual and group;  3. Peers;  4. 6-12 weeks after exposure. |
| Kenardy et al. (1996) | | 1. 2 groups (debriefing vs. assessment only);  2. No randomisation;  3. Pre- and post-data. | Earthquake | 195 | 63 / 132 | undisclosed | | Disaster relief workers | | Stress debriefing | | IES; GHQ; self-reported evaluations | Over a third of participants evaluated the debriefing to be "somewhat helpful"; those evaluations had no relation to scores on mental health outcomes; there was an overall decrease in IES scores over time but there was no significant difference between groups, on IES or GHQ, p > .05. | | | 1. Multi-session;  2. Group;  3. Mental health professional;  4. 2-years after exposure. |
| Leonard & Alison (1999) | | 1. 2 groups (CISD vs. assessment only);  2. No randomisation;  3. Pre- and post-data. | Shooting incidents | 60 | 0 / 60 | undisclosed | | Police officers | | CISD | | Coping Scale; STAXI | There were no statistical differences between groups on coping, p = .34; the CISD group had higher scores on the active coping and positive reinterpretation subscales; the CISD group scored lower on STAXI compared to control, p = .017; the less satisfied an officer was with dept. support, the angrier they felt. | | | 1. Single-session;  2. Group;  3. Trained support workers;  4. 72-hours after exposure. |
| Macnab et al. (1998) | | 1. 2 groups (CISD vs. assessment only);  2. No randomisation;  3. Pre- and post-data. | Air accident | 39 | undisclosed | undisclosed | | Healthcare professionals | | CISD | | IES; GHQ; sickness absence; self-reports | At the 6 month follow-up, there was a trend towards less severe stress symptoms in the CISD group, p = .07; however on most measures (incl. sickness absence) there were no significant differences; after 2 years, 83% reported feeling "back to normal" but many of the staff were still affected by the incident even after receiving CISD. | | | 1. Multi-session;  2. Group;  3. Trained support workers;  4. 24-48 hours after exposure. |
| Macnab et al. (2004) | | 1. 3 groups (mild vs. moderate vs. severe incidents);  2. No randomisation;  3. Post-data only. | Unexpected death of patient | 12 | undisclosed | undisclosed | | Emergency Medical Technicians | | CISD | | SASRQ; LIS; SRE; IES | There was no correlation between the severity of the incident and scores on SASRQ, LIS, SRE, or IES; and there was no change in scores over time in any of the category groups. | | | 1. Single-session;  2. Individual;  3. Trained support workers;  4. Undisclosed. |
| Matthews (1998) | | 1. 3 groups (CISD vs. assessment only);  2. No randomisation;  3. Post-data only. | Assaulted by clients | 63 | 49 / 14 | undisclosed | | Psychiatric workers | | CISD | | IES; ETRI; self-report evaluations | CISD group reported significantly higher levels of distress at time 1, compared to the control group, p = .01; In all groups, distress scores decreased over time, p < .01; there was no difference between the CISD and control groups; the CISD group reported higher levels of work stress related to traumatic exposure compared to the control group, p = .02; the CISD group had significantly higher ratings on individual measures of PTSD symptoms; 57% of those in CISD group felt that the debriefing helped reduce PTSD symptoms. | | | 1. Single-session;  2. Group;  3. Trained support workers;  4. 1-week after exposure. |
| Mitchell, Stevenson, and Poole (2000) | | 1. 1 group (no control);  2. No randomisation;  3. Post-data only. | Varied in severity (threats to person to sudden death) | 612 | 54 / 558 | 37 | | Police officers | | CISD | | Self-reported evaluations | In the first 10 weeks since the incident, 20% reported no PTSD symptoms, compared to 6.8% who reported high occurrence; 11 weeks - a year, 21.6% none, 14.8% high; 56 weeks - 2 years later, 35% no, 10.8% high; 106 weeks - 5 years, 34% none, 5.1% high; 264 weeks - 10 years, 31.3% none, 4.3% high; 530 weeks to 30 years, 30.6%, none, 6.2% high; of those who had attended CISD before, 40% recalled it as positive; following CISD 71% described it to be positive compared to only 2 as negative. | | | 1. Single-session;  2. Individual or small group;  3. Trained support workers, Managers;  4. 24-72 hours after exposure. |
| Robinson & Mitchell (1993) | | 1. 2 groups (emergency responders vs. welfare staff);  2. No randomisation;  3. Post-data only. | Varied in severity (serious injury of colleague to child fatality) | 172 | 67 / 105 | undisclosed | | Emergency Medical Technicians | | debriefing | | Self-reported evaluations | Both groups reported a reduction in the impact of the event following debriefing, p <.001; emergency responders showed greater gains than hospital staff, p <.004; all staff evaluated debriefing to be of considerable value to themselves and others; for emergency responders, the greater the impact, the more they valued the debriefing, p < .05; 96% of responders vs. 77% of welfare staff experienced a reduction in stress symptoms which they attributed to the debriefing; 34 staff reported feeling greatly affected post-debriefing. | | | 1. Single-session;  2. Group;  3. Peer;  4. 24-72 hours after exposure. |
| Palgi et al. (2012) | | 1. 1 group (no control);  2. No randomisation;  3. Post-data only. | Peacekeeping in a conflict zone | 13 | 11 / 2 | 23, 6 | | Military personnel | | Group debriefing | | IES; Self-reported evaluations | During ongoing conflict, there was a significant reduction in post-traumatic symptoms following debriefing, p =.033, d = 1.18; after a period of ceasefire followed by re-ignition of conflict, debriefing continued to reduce post-traumatic symptoms over time, p = .028, d = 1.21; across the entire period of conflict, post-traumatic symptoms decreased steadily following debriefing, p =.017. | | | 1. Multi-session;  2. Group;  3. Mental health professional;  4. 1-week after exposure. |
| Regehr & Hill (2001) | | 1. 2 groups (debriefing vs. assessment only);  2. No randomisation;  3. Post-data only. | Varied in severity (violence to self to child fatality) | 164 | undisclosed | 37.5 | | Fire Service Personnel | | Group debriefing | | BDI; IES; self-reported evaluations | 80% indicated that they found the debriefing 'helpful'; those who attended debriefing had marginally higher scores on the intrusion subscale (IES) than those who did not, p = .07; there was no difference of debriefing vs. control on BDI scores, p = .27; there was no significant correlations between subjective perceptions of the efficacy of debriefing and IES or BDI scores. | | | 1. Single-session;  2. Group;  3. Peer;  4. Undisclosed. |
| Rick et al. (2006) | | 1. 4 groups (defusing vs. SPoT vs. formal counselling vs. assessment only);  2. No randomisation;  3. Post-data only. | Varied in severity (dog attack to armed robbery) | 837 | undisclosed | 44 | | Royal Mail workers | | debriefing | | IES; GHQ; TSQ; physical/emotional functioning; social support | No significant differences were found between intervention and non-intervention groups on the IES or any subscale at follow-up; all groups showed significant health gains over time; those who received formal counselling had significantly higher rates of absences; compared to intervention, perceived organisational support was negatively correlated with absence rates, p<.01; path analysis revealed that perceptions of organisational support influenced symptom levels at 3 months post trauma and, through that, absence levels 12 months post trauma. | | | 1. Single-session;  2. Individual;  3. Manager;  4. 2-weeks after exposure. |
| Ruck et al. (2013) | | 1. 2 groups (CISD vs. assessment only);  2. No randomisation;  3. Pre- and post-data. | Varied in severity (physical assault to suicide) | 91 | undisclosed | undisclosed | | Prison staff | | CISD | | IES; GAD | At time 1, participants in the debrief group had significantly higher IES scores than in the control group, p<.01; IES scores reduced over time following debriefing, p<.001, whereas in the control group there was no difference, p>.05; descriptively, IES scores in the control group increased over time; there were no significant differences between the groups or time points on any of the GAD subscales; descriptively, GAD scores reduced following debriefing but increased in the control group. | | | 1. Single-session;  2. Group;  3. Peer;  4. 1-month after exposure. |
| Rudd et al. (2015) | | 1. 2 groups (CBT vs. TAU);  2. Random allocation;  3. Post-data only. | Peacekeeping in a conflict zone | 152 | 19 / 133 | 27.4, 6.22 | | Military personnel | | CBT | | SASI; BSSI; BDI; BAI; BHS; PCL | Soldiers who received brief CBT were approximately 60% less likely to make a suicide attempt than soldiers receiving TAU, p =.02; effects remained even after controlling for other risk factors (previous attempts, depression, stress, hopelessness, and suicidal ideation); those who previously attempted suicide, were also significantly less likely to try again following CBT, compared to TAU, p = .03; there were no between-group differences on secondary measures (BDI, BAI, BHS, PCL), ps > .10. | | | 1. Single-session;  2. Individual;  3. Trained instructor;  4. Undisclosed. |
| Shalev et al. (1998) | | 1. 1 group (no control);  2. No randomisation;  3. Post-data only. | Peacekeeping in a conflict zone | 39 | undisclosed | 19.4, 1.8 | | Military personnel | | Group debriefing | | IES; STAI; PDEQ; PSS-F; SELF-C; EXP; CEV. | There was a significant reduction in STAI scores following debriefing, p = .02, an increase in SELF-C scores, p = .001; participants who expressed high levels of anxiety before debriefing normalised after it. | | | 1. Single-session;  2. Group;  3. Mental health professional;  4. 48-72 hours after exposure. |
| Shoval-Zuckerman et al. (2015) | | 1. 2 groups (debriefing vs. assessment only);  2. No randomisation;  3. Pre- and post-data. | Peacekeeping in a conflict zone | 166 | 0 / 166 | undisclosed | | Military personnel | | Stress debriefing | | EXP; PCL; POAMS-TV; Self-rated Health; Anxiety and Defensiveness; | Participants in the control group reported more PTSD symptoms than in the debriefing group before and after the intervention, p <.05; however there was no improvement following debriefing, while in the control group, symptoms increased significantly, p < .01; following the intervention, debriefed participants were significantly better functioning than those in the control group, p <.01. | | | 1. Single-session;  2. Group;  3. Mental health professional and manager;  4. 3-months after exposure. |
| Tehrani et al. (2001) | | 1. 1 group (no control);  2. No randomisation;  3. Pre- and post-data. | Rail accident | 12 | undisclosed | undisclosed | | Supermarket employees | | Group debriefing | | IES; GAD | Four months after the group debriefing session, participants reported significant reductions in Avoidance, Arousal, and Re-experience, ps < .001, and in Anxiety and Depression, p = .003 and p <.001 respectively; indicators revealed that all employees demonstrated an improvement in job performance and reductions in the level of sickness absences; the management team reported satisfaction at having use of the occupational health team. | | | 1. Single-session;  2. Group;  3. Research team;  4. 1-week after exposure. |
| Tuckey & Scott (2014) | | 1. 3 groups (CISD vs. psychoeducation vs. assessment only);  2. Randomly allocated;  3. Pre- and post-data. | Motor vehicle accidents | 67 | 6 / 61 | undisclosed | | Fire Service Personnel | | CISD | | IES; K10; QoL; Alcohol consumption | There were no significant differences between intervention groups on IES, K10, or QoL, Fs < 1; after controlling for pre-intervention scores, the intervention had no effect on post-intervention levels of IES or K10 scores; relative to assessment (but not psychoeducation), CISD was associated with less consumption one month following intervention; relative to psychoeducation (but not assessment), CISD was associated with better QoL, post-intervention. | | | 1. Single-session;  2. Group;  3. Mental health professional and peer;  4. 72-hours after exposure. |
| Waelde et al. (2017) | | 1. 1 group (no control);  2. No randomisation;  3. Pre- and post-data. | Natural disaster | 68 | 51 / 17 | 37.3, 11.6 | | Disaster mental health workers | | Mindfulness | | disaster exposure; SI-PTSD; anxiety; CES-D; STAI | More minutes of mindfulness practice was associated with lower depression severity at follow-up, p = .02; total minutes of practice was not a predictor of anxiety, p > .05; not enough participants responded to the items about traumatic stress symptoms for them to be meaningfully analysed. | | | 1.Multi-session;  2. Group;  3. Research team;  4. 12-weeks after exposure. |
| Wu et al. (2012) | | 1. 3 groups ('PIM' vs. CISD vs. assessment only);  2. Randomly allocated;  3. Post-data only. | Natural disaster | 2,368 | undisclosed | 20.03, 3.67 | | Military personnel | | Group debriefing | | SI-PTSD; HADS | Severity of PTSD decreased over time in all 3 groups, p <.01; there was a main effect of group, p <.01; but no interaction of time*group, p =.21; PTSD scores were not significantly different at 1 month follow-up, p =.25, but were significantly lower in the PIM group than in the other two at 2 months, p <.01, and 4 months, p <.01; no differences were found between CISD and assessment only, p > .10; HADS scores decreased in all 3 groups over time, p <.01. | | | 1. Single-session;  2. Group;  3. Mental health professional;  4. 1-month after exposure. |
| Young & Parr (2004) | | 1. 2 groups (CISD vs. assessment only);  2. No randomisation;  3. Post-data only. | Varied in severity (officer-involved shooting to terrorist event) | 37 | undisclosed | undisclosed | | Police officers | | CISD | | BDI; IES; | There was no significant difference between CISD vs control on BDI, but findings trended towards improvement following CISD, p =.104; there were also no treatment effects on Avoidance or Intrusion subscales of the IES, ps >.10, but scores trended in favour of CISD; in the CISD condition, discussing job-related stressors appeared to benefit the officers by building "a sense of group cohesion". | | | 1. Multi-session;  2. Group;  3. Peer;  4. Undisclosed. |
| Note: Effect sizes are reported where available. The column titled 'Delivery' refers to how the intervention was delivered in terms of: 1. The number of sessions used; 2. The format; 3. The identity of the facilitator; and 4. The length of time since traumatic exposure. | | | | | | | | | | | | | | | | |
|  |  | |  |  |  |  |  | |  | |  | | |  |  | |
|  |  | |  |  |  |  |  | |  | |  | | |  |  | |
|  |  | |  |  |  |  |  | |  | |  | | |  |  | |

Table 2. Overview of interventions.

| **Study citation** | **Details of intervention** | **Quality appraisal score (%)** |
| --- | --- | --- |
|  |  |  |
| Adler et al. (2008) | 47-148 minute class led by trained peer instructor. Stress Management Class is comparable to stress education classes conducted by trained peers and adapted for use in a deployed environment. The course aims to introduce the concept of stress; define major and minor stressors; provide examples of deployment-related stressors; review symptoms associated with stress; identify potential long-term effects of stress on individuals; and describe adaptive coping strategies for reducing the effects of stress. | 62.96 |
| Adler et al. (2009) | 7-day programme of group debriefings led by formally trained instructors. Battlemind training takes a cognitive and skills-based approach to educating military personnel about post-deployment transition; emphasizes safety, relationships, and common physical, social and psychological reactions to combat; positively reframes traditional post-deployment transition difficulties such as PTSD, depression, and sleep problems as being a natural consequence of having developed effective occupational coping skills related to combat. | 77.78 |
| Armstrong et al. (1998) | 35-minute session conducted either one-on-one or in groups. Multiple Stress Debriefing is designed to provide services to personnel who experienced more than one incident. The aims are to encourage disclosure by participants of troubling events they experienced during the disaster relief effort; facilitate expression of associated thoughts and feelings about the troubling events; encourage discussion and the use of effective coping strategies, as well as educating participants about typical stress reactions; facilitate discussion of positive or meaningful accomplishments during the disaster relief effort while preparing participants for the transition back to their home environment. | 33.33 |
| Beaumont et al. (2016) | 12-week programme consisting of group sessions, lasting 60-90 minutes, led by a mental health professional. Compassion-focused therapy aims to help individuals learn to respond to self-criticism by accessing the contentment/self-soothing system; to cultivate a compassionate mind and learn to develop understanding for the suffering they feel; to develop compassionate attributes which include, being motivated to care for and alleviate distress (care for well-being), having a sensitivity to distress, responding to suffering with empathy, to tolerate difficult emotions (distress tolerance) and responding to distress with non-judgement. | 51.85 |
| Becker et al. (2009) | 9–12 session individual therapy led by a mental health professional. Exposure therapy aims to educate about common reactions to trauma; retrain breathing (relaxation training); prolong (repeated) exposure to trauma memories; repeat in vivo (i.e., real life) exposure to situations that you are avoiding due to trauma-related fear; encourage confronting the traumatic memory by repeatedly telling the story to the therapist and challenging things that are avoided (i.e., driving a car, walking home at night). You will be assigned homework to encourage you to practice in life the things you learn in therapy. | 37.04 |
| Belton (2017) | 7-day programme of mandatory briefings on a variety of topics such as safety, post-deployment finances, and equal opportunity rights, as well as medical screenings group sessions led by a trained peer instructor. The debriefing model included a strong narrative element of reconstructing the story of the event(s), encouraging good mental and physical health, well-being, work satisfaction, and enjoyment of life, both during active duty military service and continuing after military service. | 62.96 |
| Biggs et al. (2016) | 2-hour group session led by a mental health professional. The intervention was based on the principles of Psychological First Aid the aims for which were to reduce distress and facilitate adaptation by promoting five principles: (a) safety (physical and psychological), (b) calming, (c) connectedness, (d) self-efficacy, and (e) hope/optimism. | 55.56 |
| Blacklock (2012) | 90-minute group defusing/debriefing session led by trained peer instructors. The intervention's aims were to invited staff to describe their role in the incident; to describe the thoughts they experienced; gently move from a cognitive to emotional level by thinking about the worst aspects of the incident; move back to a more cognitive level by describing the physical symptoms during and after the incident; emphasize the normality of dealing with an abnormal event; teach strategies for dealing with stress; provide details of additional support services. | 40.74 |
| Brandt et al. (1995) | A multi session group-oriented debrief. The aim of the intervention was for participants to share their experiences of a common trauma. | 25.93 |
| Carlier et al. (1998) | A 60-minute group based debrief led by trained peer instructors and mental health professionals. The aims of the intervention were to (1) introduce the ground rules; (2) establish what happened; (3) discuss thoughts about what happened; (4) discuss emotions associated with the event; (5) identify signs and symptoms of distress; (6) emphasize normality and furnish information about useful coping strategies and about the post-traumatic stress syndrome in general; and (7) discuss any remaining issues, to summarize and to provide any additional services. | 55.56 |
| Carlier et al. (2000) | A multi-session one-on-one debrief, averaging 74-minutes in total, led by trained peer instructors. Based on the principles of CISD, this intervention was adapted for use with individual trauma victims. The debriefer applied a seven-stage, semi-structured procedure, comprising: an introduction; facts; thoughts and impressions; emotional reactions; normalization and traumatic stress education; planning for the future; and disengagement. The debriefing itself focused solely on the critical incident and its effects, and it was supplemented by written documentation that explained common reactions to traumatic events; suggested strategies to deal with such reactions; and gave contact details if further immediate help was required. | 51.85 |
| Chemtob et al. (1997) | A 3-hour group debriefing (followed by 2-hour lecture) led by a trained instructor. The aim of the intervention was to achieve three goals of normalization, education, and psychological support. Participants were then invited to share with the group their experiences. The immediacy and intensity of experiences made sharing reactions easy. The facilitator invited a description of the experience touching on its cognitive, affective, and behavioural components. Description of the most terrifying moments was also invited. The facilitator encouraged and acknowledged the intensity of the experience and emphasized the universality of reactions and the fact of competent survival. Perceived failure was reframed as something to be expected in a disaster. | 37.04 |
| Chongruksa et al. (2012) | 3-month group intervention, each session lasting 1.5-2 hours, led by a trained research team. The intervention comprises 8 sessions that aim to1) educate about mental health, instruct on deep breathing exercises ; 2) foster trust; exchange stories of distress; 3) encourage discussion about anxiety; 4) monitor anxieties and challenge dysfunctional thoughts; 5) exchange stories of anger and modify angry thoughts and perceptions; 6) encourage disclosure of feelings of guilt and sadness; to reflect upon impermanence and practice mindfulness; 7) brainstorm ways to achieve thought stopping; 8) review and summarise ideas; 9) share stories of achievement; 10) make plans to achieve unmet goals; 11) and 12) exchange stories of enacting plans, evaluate current behaviour and encourage commitment. | 66.67 |
| Chongruksa et al. (2015) | 2-day programme of group therapies, each session lasting 50 minutes to 1.5-hours, led by a trained research team. The intervention consisted of 3 phases, the first was intended to help members understand the groups’ goals, leaders’ and members’ roles, to build relationship and trust, to understand information on mental health and resilience, to begin building their philosophy of living and to teach team work, to self-disclose and to practice breathing. The second phase aimed at increasing resilience and mental health, exchanging experiences of deployment, discussing hardship, finding meaning in their careers, sharing life goals and learning approaches to optimism. The third phase was to continue to increase resilience and mental health, encourage members to believe in themselves, increase their self-esteem and become more aware of positive, negative and neutral thoughts. | 70.37 |
| Cigrang et al. (2005) | 5-week programme of individual debriefing sessions, lasting around 90-minutes each, led by a mental health professional. The intervention included 4 sessions of exposure therapy in which the primary focus was prolonged imaginal exposure to the traumatic event. Individuals described the incident step-by-step in concrete detail; the setting prior to the event; what they saw; what they were wearing; smells, aromas...etc; to speak aloud anything they said. The instructors then reassured participants that it was not uncommon for people to become upset during the exposure or feel similar to how they felt during the incident itself. They were encouraged to not be embarrassed by their reactions but see them as normal responses to an abnormal event. Brief cognitive therapy was included in the session to provide strategies to reframe negative or intrusive thoughts. | 22.22 |
| Cigrang et al. (2017) | 4-6 week programme of individual therapy sessions led by a mental health professional. The BHC educated the patient about factors that contribute to the development and maintenance of PTSD symptoms, with an emphasis on the role of avoidance. The appointment concluded with the BHC presenting PTSD treatment options, which included (a) meeting with the BHC in primary care for four 30-min appointments; (b) referral to the specialty mental health clinic for more intensive, evidence-based psychotherapy for PTSD; and (c) addressing the symptoms using self-help resources only. | 70.37 |
| Deahl et al. (1994) | 1-day session of psychological debriefing led by welfare professionals (chaplains, psychologist, psychiatrists or social workers). The intervention included an educational component, in which the symptoms of post-traumatic stress were explained as a normal human reaction to abnormal stress, a small group debriefing session with two welfare professionals, using the Dyregov (1989) model, and formal advice on where to get help, if required. The emphasis was on the frequency and normality of any disturbing symptoms, in an attempt to destigmatise and facilitate help-seeking. | 37.04 |
| Deahl et al. (2000) | 2-hour debriefing session led by a trained researcher. The intervention follows the principles of the Dyregov and Mitchell model, seeking to promote emotional processing and ventilation by encouraging the recollection and reworking of the traumatic event. | 74.07 |
| Dickstein et al. (2013) | 12-part programme of 50-60 minute, one-on-one sessions led by a mental health professional. The primary focus of the intervention is to re-frame negative thoughts after being exposed to a traumatic event. The first phase consists of psychoeducation, outlining the cognitive theory of PTSD development; the next phase involves formal processing of the incident; to modify cognitive distortions. The final phase focuses on reinforcing skills that participants learn to further identify, evaluate, and modify beliefs concerning trauma. Additional sessions could be added at the end of therapy, as needed, to address remaining symptoms. | 62.96 |
| Difede et al. (2007) | 12 CBT sessions, lasting 75-minutes each, led by a mental health professional. The intervention included: (a) psychoeducation; (b) treatment rationale and contracting; (c) breathing exercises; (d) imaginal exposure; (e) gradual in vivo exposure; (f) cognitive reprocessing; (g) relapse prevention; and (h) homework (imaginal exposure using audiotaped imaginal exposure from treatment sessions, graduated in vivo exposure, and cognitive reprocessing). Patients were instructed to do their homework daily. Twelve 75-minute sessions were held weekly. Imaginal exposure was begun in the second session with a target of 30 minutes per session and continued through the 11th session. | 92.59 |
| Drury et al. (2013) | Participants expressed strong desire to learn how to recognise stress in themselves and in their colleagues; sought guidance on helping colleagues who are stressed; peer support programmes; training in listening skills and a number of other simple psychosocial techniques for peer supporters. | 37.04 |
| Firing et al. (2015) | 1-2 hour interview led by a trained researcher. This consisted of three main sections: Stage 1, getting the informants to describe their experience of the incident. Stage 2, the main part of the interview guide consisted of questions exploring the team's experience of the debriefing process. The researcher was flexible about changing the order of questions and probed areas that arose in order to follow the participants’ perspectives. Stage 3, using sufficient time in bringing the interview to an end. This was important as it helped to maintain the trustful climate between the informant and the researcher. Holistic Debriefing benefits from traditional debriefing, the “after action review,” and from psychological debriefing, by explicitly addressing and including emotional aspects. | 44.44 |
| Frappell-Cooke et al. (2010) | A single-session group debrief, led by trained peer supporters. The aim of the intervention was to provide: basic psychoeducation; appropriate feedback and support following traumatic exposure; recognition of psychological illness; understanding and acceptance of stress reactions within an appropriate environment. | 51.85 |
| Greenberg et al. (2010) | 1-2 day peer-delivered psychological support process, which aims to ensure that those who develop psychological disorders as a result of being exposed to traumatic events, are assisted to seek help. | 59.26 |
| Grundlingh et al. (2017) | 90-120 minute group debriefings led by a mental health professional. The intervention involved story-telling, identifying emotional responses to these stories, psycho-education and practical information to normalise group member reactions to a distressing event. Session 1 focused on encouraging group participation, discussing primary trauma encountered and emotional reactions to these stories. Session 2 connected current experiences with the group members’ own trauma histories and life experiences. The last session focussed on societal and community responses to violence, and employing personal agency to find constructive ways to address violence in communities. | 81.48 |
| Gunasingam et al. (2015) | 1-day, multi-phase group debrief led by a senior mental health professional. Each session started with the facilitator asking each participant how the previous fortnight had been and then allowing the topics for discussion to emerge generically from their concerns and experiences. Facilitators had topics of themes to raise if no participant had anything to say. Facilitators kept a log of the themes covered. | 74.07 |
| Halpern et al. (2009) | 1-2 hour debrief led by peer supporters. The intervention focuses on providing emotional support by the supervisor, which consists of: acknowledgment of the incident as critical, valuing the work done by the emergency responder; showing concern about the well-being of the emergency responder, willingness to listen and to offer material help. The intervention also ensures availability of a brief (often just 1/2–1 h, rarely more than a few hours) timeout period, usually spent in what appears to be casual conversation with peers, but which serves to decrease emotional hyperarousal and allows for self-titrated release of emotion, in the context of a comfortable, understanding environment. | 40.74 |
| Harris et al. (2011) | A 1-hour group debrief led by an experienced researcher. The intervention followed the structure of CISD, providing psychoeducation on various stress reactions following exposure to a critical incident. The seven phases of CISD are labelled as follows: (a) introduction, (b) fact, (c) thought, (d) reaction, (e) symptoms, (f) teaching, and (g) re-entry to the workplace. | 59.26 |
| Hunt et al. (2013) | A single-session, one-on-one interview and debriefing led by a trained peer supporter. The briefings aimed to provide high-quality information about potential psychological trauma responses and self-management. Following a structured psychological risk assessment, those considered at heightened risk of developing trauma-related mental health problems are signposted to self-refer to appropriate help sources. | 66.67 |
| Hutton et al. (2010) | A 1-3 hour, group debrief based on the principles of CISD led by a trained instructor. Bereavement debriefing sessions focus on the emotional response of health care professionals, often in the wider context of a relationship with the patient and not simply the death event itself. The intervention follows the structure of 1) Welcoming and introducing the sessions; 2) reviewing facts of the incident; 3) reviewing the patient case; 4) eliciting responses to the incident; 5) discussing the emerging emotions; 6) discussing coping strategies for grief; 7) discussing lessons learned; and 8) concluding the session, acknowledging the care and support available for staff and families. | 37.04 |
| Jones et al. (2017) | A multi-session, one-on-one structured interview, followed by small group debriefing led by a trained practitioner. The intervention follows a structure of: 1) Carrying out effective psychological management at the site of a traumatic event; 2) Convening and conducting a meeting with key unit managers to plan a response; 3) Analysing traumatic events and allocating personnel to group or individual risk assessment; 4) Conducting a risk-assessment interview; 5) Conducting a briefing meeting; 6) Facilitating a timely referral to an appropriate agency for treatment. | 55.56 |
| Kenardy et al. (1996) | A 3-5 hour group debriefing led by a mental health professional. The intervention followed the 7 phases of CISD: introductory phase (rules and process explained); fact phase (what they saw, heard, smelled, touched and did); thoughts phase (first thoughts); feelings phase (emotional reactions); assessment phase (physical or psychological symptoms); education phase (stress response syndrome); and re-entry phase (referral information). | 40.74 |
| Leonard & Alison (1999) | A 30 minute group debrief led by a mental health professional and peer supporters. The intervention followed the 7-phase process of CISD. Throughout each section individuals are, first, encouraged to ventilate strong feelings aroused by the incident. Second, each police officer is then reassured that such strong feelings are perfectly normal. Third, the person is cautioned that some symptoms have a delayed occurrence. Finally, attempts are made to help the person to assimilate the experience and see it in context. | 62.96 |
| Macnab et al. (1998) | A 3-4 hour group debrief led by a trained instructor and assisted by critical incident stress management staff. Following the principles of CISD, the intervention aimed to provide information concerning the symptoms of critical stress reactions and strategies for coping with them. The sessions allowed participants to relate their involvement in and their reactions to the event to each other, and to hear the perspectives of others. | 44.44 |
| Macnab et al. (2004) | A 15-45 minute, one-on-one debrief which included immediate debriefing (at the scene), initial defusing (within hours), formal debriefing (within days), and follow-up debriefing. The intervention ranged from "mild" to "severe", depending on a severity scoring system. Mild intervention was a "listening ear" over the telephone and direction to a pamphlet describing the symptoms of the critical incident stress (pamphlets were sent to all stations). A moderate intervention was a "listening ear" immediately, direction to the pamphlet, and referral to a critical-incident stress coordinator for debriefing. A severe intervention was relevant only if more than one person involved in an event experienced CIS. A severe intervention consisted of defusing and subsequent debriefing with a critical incident stress coordinator. | 37.04 |
| Matthews (1998) | A single-session group debrief led by a qualified support worker. A key component of the debriefing process was the emphasis on restoration of the individual's coping skills and promotion of a sense of control after the event. Participants also co-developed a cognitive-psychological framework from which an understanding of their reactions and the recovery process could be gained. Participants were encouraged to discuss facts of the event, clarify questions, and explore thoughts, feelings, and coping strategies. | 51.85 |
| Mitchell, Stevenson, and Poole (2000) | A 1-3 hour one-on-one or small group debriefing led by a trained instructor. The intervention took the form of a structured group discussion with facilitators and the individuals who were involved in the incident. The aim was to review the facts, thoughts, impressions and reactions following a critical incident; to provide information on normal reactions to abnormal events; and to identify those persons who may require more intensive psychological support. | 40.74 |
| Robinson & Mitchell (1993) | 2-3 hour group debrief led by trained peers. The intervention follows the principles of CISD, providing the opportunity for a discussion about an incident, or series of incidents, with focus on how personnel have managed and currently are coping. Through a confidential and supportive environment, members are able to discuss issues of concern to them and to support others in the group. | 40.74 |
| Palgi et al. (2012) | A multi-session, group debrief led by mental health professional. The approach emphasizes jumping narratively between the past and the future in order to treat the trauma in the present. More specifically, the model is based on the memory's plasticity, first shifting the traumatic event back into a continuation of an individual’s past narrative about the occurrence, and then integrating it with a narrative of the future. This is done without making the substantive core of the experience of the traumatic event—called the “traumatic nucleus”—the major focus of treatment, as is done in mainstream, exposure-oriented models of treatment. | 48.15 |
| Regehr & Hill (2001) | A single-session group debrief led by a trained peer instructor. The first stage is to review the event. This includes describing the sights, sounds and smells associated with the event, discussing each individual’s involvement and the final outcome. Next, people are invited to discuss their reactions to the event including the emotional and behavioural consequences both for themselves and their family life. Following this, the group leader provides educational information designed to normalize reactions and reinforce coping skills. As the session draws to a conclusion, participants are invited to discuss their accomplishments and reinforce one another’s efforts. Finally, participants are encouraged to provide mutual aid as required and opportunities for professional follow-up are presented. | 29.63 |
| Rick et al. (2006) | 90-minute one-on-one debrief led by a trained peer supporter. The debrief itself involved participants giving their account of the traumatic incident, looking at the facts of the incident and any thoughts and feelings associated with the incident. The final phase of the debrief is the provision of information. The information includes outlining normal reactions to trauma, providing some guidance and simple hints on how to reduce or prevent traumatic reactions, followed by a closing stage during which any remaining questions are answered. | 48.15 |
| Ruck et al. (2013) | 2-3 hour group debrief led by a trained peer instructor. The intervention followed the principles of CISD including introductions (roles within incident), facts (details of incident), thoughts (during incident), feelings (emotions and sensations during incident), reactions (since incident to present), planning (future issues, e.g. inquiries, investigations) and closure. | 55.56 |
| Rudd et al. (2015) | 12-week programme of 90-minute, one-on-one psychotherapy sessions led by a mental health professional. The intervention consisted of 3 phases: 1) A detailed assessment of the participant’s most recent suicidal episode or suicide attempt; 2) application of strategies to reduce beliefs or assumptions that serve as vulnerabilities to suicidal behaviour (guilt and shame); 3) a relapse prevention task wherein participants imagined the circumstances of a previous episode and the internal experiences associated. Then participants imagined themselves using one or more of the skills learned in CBT to resolve the crisis. | 96.30 |
| Shalev et al. (1998) | 2.5-hour group debrief led by a mental health professional. Debriefing sessions were conducted immediately after combat, in the presence of all the survivors. The group's task was defined as "describing the combat with all the possible details." Military ranks were set aside during the session, and testimonies were weighed according to their pertinence to understanding the course of the operation. The reconstruction of the battle followed a "strict chronological path" and uncovered the events in sequential order. Information on each stage of the action was exhaustively collected from all witnesses. Importantly, soldiers' thoughts, assumptions, and feelings and the resulting decisions were considered to be an important part of the factual reality of combat. | 51.85 |
| Shoval-Zuckerman et al. (2015) | A 6-hour group debrief led by a mental health professional. The intervention consisted of three parts: In the first part, the soldiers reviewed the sequence of events that occurred from the time of their release from army reserve duty until the present. In the second part, they articulated their thoughts and feelings at the present time. They were given an opportunity to relate to the losses they had experienced in the war, and they were able to express feelings of guilt and anger. In the third part, the soldiers discussed their ability to continue functioning as individuals and as a group. The discussion focused on the need to continue living, and the expectation that participation in the group would enable them to resume regular functioning. Emphasis was placed on the strength of the group and the positive coping mechanisms used. | 70.37 |
| Tehrani et al. (2001) | 1-day group debrief led by trained instructors. The debriefing went through the employees’ experiences of the incident in great detail, focusing on their behaviours and the things that were seen, heard, smelled, touched and tasted. The debriefing allowed the employees to examine what had happened during the day of the crash, to fill gaps in the story, check understanding and share knowledge. Of particular significance were the changes in attitude that occurred during the debriefing. At the beginning of the debriefing, the participants talked about those things that they would have liked to have done but did not do. At the end of the debriefing, colleagues were recognizing the positive things that they had achieved individually and as a group. This process allowed for the group to create a shared understanding of what had happened. | 51.85 |
| Tuckey & Scott (2014) | 90-minute group debrief led by a mental health professional. The intervention followed the seven-phase protocol of CISD: (1) Introduction, (2) Facts, (3) Thoughts, (4) Reactions, (5) Symptoms, (6) Education, and (7) Re-entry. Sessions were led by a consultant mental health professional. A peer supporter also attended and led the introduction and education phases. | 81.48 |
| Waelde et al. (2017) | 4-hour group workshop led by a trained instructor. The standard implementation is an 8-week group-based manualized and secularized mindfulness meditation and mantra intervention with a booster session after 12 weeks. This standard format was modified for the disaster context to include an initial workshop and home study program. The workshop included instruction and guided practice in the mindfulness techniques such as encouraging participants to notice their breathing as often as possible, particularly during stressful moments. It also provided information about how to teach the practices to disaster survivors. The home program used a manual which provided week-by-week guidance designed to promote learning and daily practice. | 66.67 |
| Wu et al. (2012) | 2-hour group debrief led by a mental health professional. The intervention followed the principles of CISD including introductions (roles within incident), facts (details of incident), thoughts (during incident), feelings (emotions and sensations during incident), reactions (since incident to present), planning (future issues, e.g. inquiries, investigations) and closure. The greatest difference between ‘‘512 PIM’’ and CISD is the 5th stage which focused on cohesion training, for example participants were instructed to play games which need team cooperation, participants were asked to tell in private or shout in public the words they most want to say. | 77.78 |
| Young & Parr (2004) | A 60-minute group debrief led by a trained peer supporter. Each meeting began with the question, “What has been the most stressful or difficult part of being a police officer this week?” Each time, we went around the circle, and each officer participated in some way. At the end of each meeting, the researcher taught methods for coping with and understanding the types of stress discussed during the meeting. General information about stress and coping was also presented. In a number of group meetings, teaching occurred from officer to officer, sometimes about decision making and coping, other times about how decisions or problems had been faced by others. This interaction seemed especially effective and helpful and also built a sense of group cohesion. | 44.44 |

**Full references to studies included in this review.**

Adler, A., Litz, B., Castro, C. A., Suvak, M., Thomas, J. L., Burrell, L., McGurk, D., Wright, K. M., Bliese, P. D. (2008). A group randomized trial of critical incident stress debriefing provided to U.S. peacekeepers. *Journal of Traumatic Stress, 21*, 253-263, 10.1002/jts.20342

Adler, A., Bliese, P., McGurk, D., Hoge, C., Castro, C. (2009) Battlemind debriefing and battlemind training as early interventions with soldiers returning from Iraq: Randomization by platoon. *Journal of Consulting and Clinical Psychology, 7*, 928-940, 10.1037/a0016877

Armstrong, K., Zatzick, D., Metzler, T., Weiss, D., Marmar, C., Garma, S., Ronfeldt, H., Roepke, L. (1998). Debriefing of American Red Cross personnel: Pilot study on participants’ evaluations and case examples from the 1994 Los Angeles earthquake relief operation. *Social Work in Health Care, 27*,33-50, 10.1300/J010v27n01_03

Beaumont, E., Durkin, M., McAndrew, S., Martin, C. R. (2016). Using compassion focused therapy as an adjunct to trauma-focused CBT for fire service personnel suffering with trauma-related symptoms. *The Cognitive Behaviour Therapist, 9,* 10.1017/s1754470x16000209

Becker, C. B., Meyer, G., Price, J. S., Graham, M. M., Arsena, A., Armstrong, D. A., Ramon, E. (2009). Law enforcement preferences for PTSD treatment and crisis management alternatives. *Behaviour Research and Therapy, 47*, 245-253.

Belton, J. (2017). Soldiers’ debriefings attitudes, combat exposure, and deployment stressors on post-deployment mental health symptoms. *The Catholic University of America: Digital Collections*.

Biggs, Q. M., Fullerton, C. S., McCarroll, J. E., Liu, X., Wang, L., Dacuyan, N. M., Zatzick, D. F., Ursano, R. J. (2016). Early intervention for post-traumatic stress disorder, depression, and quality of life in mortuary affairs soldiers postdeployment. *Military Medicine, 181*.

Blacklock, E. (2012). Interventions following a critical incident: Developing a critical incident stress management team. *Archives of Psychiatric Nursing, 26.*

Brandt, G. T., Fullerton, C. S., Saltzgaber, L., Ursano, R. J., Holloway, H. (2009). Disasters: Psychologic responses in health care providers and rescue workers. *Nordic Journal of Psychiatry, 49*, 89-94, 10.3109/08039489509011889

Carlier, I., Lamberts, R., Van Uchelen, A., Gersons, B. (1998). Disaster-related post-traumatic stress in police officers: a field study of the impact of debriefing. *Stress and Health, 14*, 143-148, 10.1002/(SICI).1099-1700(199807).14:3<143::AID-SMI770>3.0.CO;2-S

Carlier, I., Voerman, A., Gersons, B. (2000). The influence of occupational debriefing on post- traumatic stress symptomatology in traumatized police officers*. British Journal of Medical Psychology, 73*, 87-98.

Chemtob, C. M., Tomas, S., Law, W., Cremniter, D. (1997). Postdisaster psychosocial intervention: a field study of the impact of debriefing on psychological distress*. American Journal of Psychiatry, 154,* 415-417, 10.1176/ajp.154.3.415

Chongruksa, D., Penprapa, P., Sawatsri, S., Pansomboon, C. (2012). Efficacy of eclectic group counselling in addressing stress among Thai police officers in terrorist situations. *Counselling Psychology Quarterly, 25*, 83-96, 10.1080/09515070.2012.666424

Chongruksa, D., Penprapa, P., Sawatsri, S., Pansomboon, C. (2015). Integrated group counselling to enhance mental health and resilience of Thai army rangers. *Asia Pacific Journal of Counselling and Psychotherapy, 6*, 41-57, 10.1080/21507686.2015.1091018

Cigrang, J., Peterson, A., Schobitz, R. (2005). Three American troops in Iraq: Evaluation of a brief exposure therapy treatment for the secondary prevention of combat-related PSTD. *Pragmatic Case Studies in Psychotherapy, 1*.

Cigrang, J., Rauch, S. A., Mintz, J., Brundige, A. R., Mitchell, J. A., Najera, E., Litz, B. T., Young- McCaughan, S., Roache, J. D., Hembree, E. A., Goodie, J. L., Sonnek, S. M., Peterson, A. L., Strong Star Consortium (2017). Moving effective treatment for posttraumatic stress disorder to primary care: A randomized controlled trial with active duty military. *Families, Systems and Health, 35*, 450-462, 10.1037/fsh0000315

Deahl, M., Gillham, A., Thomas, J., Searle, M., Srinivasan, M. (1994). Psychological sequelae following the gulf war factors associated with subsequent morbidity and the effectiveness of psychological debriefing. *British Journal of Psychiatry, 65,* 60-65,

Deahl, M., Srinivasan, M., Jones, N., Thomas, J., Neblett, C., Jolly, A. (2000). Preventing psychological trauma in soldiers: The role of operational stress training and psychological debriefing. *Psychology and Psychotherapy, 73*, 77-85, 10.1348/000711200160318

Dickstein, B. D., Walter, K. H., Schumm, J. A., Chard, K. M. (2013). Comparing response to cognitive processing therapy in military veterans with subthreshold and threshold posttraumatic stress disorder. *Journal of Traumatic Stress, 26*, 703-709, 10.1002/jts.21869

Difede, J., Malta, L. S., Best, S., Henn-Haase, C., Metzler, T., Bryant, R., Marmar, C. (2007). A randomized controlled clinical treatment trial for World Trade Center attack-related PTSD in disaster workers. *Journal of Nervous and Mental Disease, 195,* 861-865, 10.1097/NMD.0b013e3181568612

Drury, J., Kemp, V., Newman, J., Novelli, D., Doyle, C., Walter, D., Williams, R. (2013). Psychosocial care for persons affected by emergencies and major incidents: A Delphi study to determine the needs of professional first responders for education, training and support. *Emergency Medicine Journal, 30*, 831-836,

Firing, K., Johansen, L., Moen, F. (2015). Debriefing a rescue mission during a terror attack. *Leadership & Organization Development Journal, 36*, 778-789, 10.1108/lodj-12-2013-0169

Frappell-Cooke, W., Gulina, M., Green, K., Hacker-Hughes, J., Greenberg, N. (2010). Does trauma risk management reduce psychological distress in deployed troops? *Occupational Medicine, 60*, 645-650.

Greenberg, N., Langston, V., Everitt, B., Iversen, A., Fear, N. T., Jones, N., Wessely, S. (2010). A cluster randomized controlled trial to determine the efficacy of Trauma Risk Management (TRiM) in a military population. *Journal of Traumatic Stress, 23,* 430-436, 10.1002/jts.20538

Grundlingh, H., Knight, L., Naker, D., Devries, K. (2017). Secondary distress in violence researchers: a randomised trial of the effectiveness of group debriefings. *BMC Psychiatry, 17,* 204, 10.1186/s12888-017-1327-x

Gunasingam, N., Burns, K., Edwards, J., Dinh, M., Walton, M. (2015). Reducing stress and burnout in junior doctors: The impact of debriefing sessions. *Postgraduate Medical Journal, 91,* 182-187,

Halpern, J., Gurevich, M., Schwartz, B., Brazeau, P. (2009). Interventions for critical incident stress in emergency medical services: A qualitative study. *Stress and Health, 25,* 139-149, 10.1002/smi.1230

Harris, M., Baloglu, M., Stacks, J. (2011). Mental health of trauma-exposed firefighters and critical incident stress debriefing. *Journal of Loss and Trauma, 7,* 223-238, 10.1080/10811440290057639

Hunt, E., Jones, N., Hastings, V., Greenberg, N. (2013). TRiM: An organizational response to traumatic events in Cumbria Constabulary. *Occupational Medicine, 63,* 549-555.

Hutton, N., Hall, B., Rushton, C. (2010). Bereavement debriefing sessions: An intervention to support health care professionals in managing their grief after the death of a patient. *Pediatric Nursing, 36*, 185-189.

Jones, N., Burdett, H., Green, K., Greenberg, N. (2017). Trauma Risk Management (TRIM).: Promoting help seeking for mental health problems among combat-exposed U.K. military personnel. *Psychiatry, 80*, 236-251, 10.1080/00332747.2017.1286894

Kenardy, J., Webster, R., Lewin, T., Cart, V., Hazeli, P., Carter, G. (1996). Stress debriefing and patterns of recovery following a natural disaster. *Journal of Traumatic Stress, 9,* 37-49.

Leonard, R., Alison, L. (1999). Critical incident stress debriefing and its effects on coping strategies and anger in a sample of Australian police officers involved in shooting incidents. *Work & Stress, 13*, 144-161, 10.1080/026783799296110

Macnab, A., J., Russell, J. A., Lowe, J. P., Gagnon, F. (1998). Critical incident stress intervention after loss of an air ambulance: Two-year follow-up. *Prehospital and Disaster Medicine, 14,* 15-19, 10.1017/s1049023x0002848x

Macnab, A., Sun, C., Lowe, J. (2004). Randomized, controlled trial of three levels of critical incident stress intervention. *Prehospital and Disaster Medicine, 18,* 367-371, 10.1017/s1049023x00001333

Matthews, L. (1998). Effect of staff debriefing on posttraumatic stress symptoms after assault by community housing residents. *Psychiatric Services, 49,* 207-212, 10.1176/ps.49.2.207

Mitchell, M., Stevenson, K., Poole, D. (2000) Managing post incident reactions in the police service. *Health and Safety Executive* .

Robinson, R., Mitchell, J. T. (1993). Evaluation of psychological debriefings. *Journal of Traumatic Stress, 6,* 367-382, 10.1007/BF00974135

Palgi, Y., Ben-Ezra, M., Possick, C. (2012). Vulnerability and resilience in a group intervention with hospital personnel during exposure to extreme and prolonged war stress. *Prehospital and Disaster Medicine, 27*, 103-108, 10.1017/S1049023X12000283

Regehr, C., Hill, J. (2001). Evaluating the efficacy of crisis debriefing groups. *Social Work With Groups, 23,* 69-79, 10.1300/J009v23n03_06

Rick, J., O'Regan, S., Kinder, A. (2006). Early intervention following trauma: A controlled longitudinal study at Royal Mail Group. *Institute for Employment Studies.*

Ruck, S., Bowes, N., Tehrani, N. (2013). Evaluating trauma debriefing within the UK prison service. *The Journal of Forensic Practice, 15,* 281-290, 10.1108/jfp-09-2012-0018

Rudd, M. D., Bryan, C. J., Wertenberger, E. G., Peterson, A. L., Young-McCaughan, S., Mintz, J., Williams, S. R., Arne, K. A., Breitbach, J., Delano, K., Wilkinson, E., Bruce, T. O. (2015). Brief cognitive-behavioural therapy effects on post-treatment suicide attempts in a military sample: Results of a randomized clinical trial with 2-year follow-up. *American Journal of Psychiatry, 172*, 441-449, 10.1176/appi.ajp.2014.14070843

Shalev, A. Y. P., Tuvia, Rogel-Fuchs, Yael, Ursano, Robert J., Marlowe, David (1998). Historical group debriefing after combat exposure. *Military Medicine, 163*, 494-498, 10.1093/milmed/163.7.494

Shoval-Zuckerman, Y., Dekel, R., Solomon, Z., Levi, O. (2015). The effectiveness of early group intervention for military reserves soldiers: The role of the repressive coping style. *The Israel Journal of Psychiatry and Related Sciences, 52,* 49-59.

Tehrani, N., Walpole, O., Berriman, J., Reilly, J. (2001). A special courage: Dealing with the Paddington rail crash. *Occupational Medicine, 51,* 93-99.

Tuckey, M. R., Scott, J. E. (2014). Group critical incident stress debriefing with emergency services personnel: A randomized controlled trial. *Anxiety Stress, and Coping, 27*, 38-54, 10.1080/10615806.2013.809421

Waelde, L., Hechanova, M., Ramos, P., Macia, K., Moschetto, J. (2017). Mindfulness and mantra training for disaster mental health workers in the Philippines. *Mindfulness, 9,* 1181-1190.

Wu, S., Zhu, X., Zhang, Y., Liang, J., Liu, X., Yang, Y., Yang, H., Miao, D. (2012). A new psychological intervention: "512 Psychological Intervention Model" used for military rescuers in Wenchuan Earthquake in China. *Social Psychiatry and Psychiatric Epidemiology, 47*, 1111- 1119.

Young, A., Parr, G. (2004). An examination of the effectiveness of periodic stress debriefings with law enforcement personnel. In G. R. Walz & R. K. Yep (Eds.), *VISTAS: Perspectives on counselling*, 2004, 145-152.
