# Supplementary material for: Early Post-trauma Interventions in Organizations: A Scoping Review
Source: Front Psychol. 2020 Jun 25;11:1176. doi: 10.3389/fpsyg.2020.01176 (PMC7330139; doi:10.3389/fpsyg.2020.01176)
Supplement: Supplementary file 3 [file Table_3.DOCX]

**Supplementary Material 3 –** Full results from the meta-ethnography.

| **Study citation** | **Adherence** | **Organisational context** | **Governance** | **Social support** | **Perceived benefits** |
| --- | --- | --- | --- | --- | --- |
|  |  |  |  |  |  |
| Adler et al. (2008) | *"we adhered to the CISD model* [with] *personnel trained in CISD"* | The sessions were comparable to stress education classes but were *"adapted for use in a deployed environment"* | *"Study personnel had no formal relationship to the soldiers or unit in the study. They were not in the chain of command or part of the deployed unit."* | The intervention places *"emphasis on peer processes"* | CISD is *"well received and well liked"* and *"appeared to have some positive effects on outcomes relevant to a military organization"* |
| Adler et al. (2009) | *"A protocol deviation did occur ... to fit the* [team] *schedule"  "adapted by military community mental health personnel"* | The intervention *"require*[d] *a tailored approach".  "for organizational reasons it was not feasible to include a condition that received no intervention"* | Platoon leaders were used to "*to reinforce this support"  "leaders are routinely expected to conduct sessions".* | *"focuses on unit cohesion, identifies what peers and leaders can do to help unit members"* | *"participants rated Battlemind debriefing and Battlemind training more positively than stress education"* |
| Armstrong et al. (1998) | The intervention outlined followed principles of CISD but differed by being designed to respond to multiple incidents and conducted 1-in-1 or in groups. | *"developed specifically to address and debrief ARC personnel "* | *"*[the organisation] *created a mental health component to provide services including debriefing to their workers"* | *"opportunity for members to support each other"  "talking with others...was the most helpful element of the debriefing"* | *"Men* [rated] *the debriefing somewhat more positively than women, and...as the number or participant to facilitators in their groups decreased."* |
| Beaumont et al. (2016) | The intervention outlined followed a previously published model. | The model was developed specifically for those in roles who experience *"self-criticism, self-blame and shame"* | Workers were referred to external psychotherapists for treatment rather than treated in-house. |  | The intervention was found to be *"acceptable"* to those experiencing symptoms of PTSD despite it have no demonstrable influence of symptom severity. |
| Becker et al. (2009) |  | Participants may have been reluctant to *"seek treatment but did not want to indicate this"*. Possibly as a result of the stigma associated with seeking help in that population. | Participants were keen for treatment to be mandated by their organisation as a means to avoid stigma and to highlight that the organisation is meeting their duty of care to workers. | Participants indicated favouring group-based treatments even if they showed no efficacy on objective markers of PTSD. | *"Exposure* [therapy] *was rated as significantly more credible, although the actual magnitude of difference was fairly small."* Medication was the least popular therapy. |
| Belton (2017) | *"group psychological debriefings in general are examined rather than any one specific model"* | *"The practicalities of accessing this population preclude*[d] *random selection"* | The study outlined refers to a mandatory set of briefings conducted at and by the organisation. | *"Positive social support, both before and after the traumatic event, is a protective factor"* | *"participants often rated the intervention favorably".* Subjective evaluations were associated with scores for symptom severity. |
| Biggs et al. (2016) | *"based on the principles of PFA"* | "*intervention efficacy may have been influenced by … number and spacing of sessions"* owing to *"command and job duties*". | The study outlined refers to an intervention conducted at and by the organisation. | *"communicating with others"* and *"providing support to a buddy"* were reported to be among the most helpful aspects of the intervention. | *"Intervention content rated as most helpful included communicating with others...having a positive outlook on...relaxation techniques...and problem solving"* |
| Blacklock (2012) | The intervention outlined, follows CISD principles but *"conjoins the two windows of response into a single session"* | *"an adapted version proved useful in this case"  "need to tailor models of CISM to institutional circumstances"* | Workers were thankful for the debriefing, in particular appreciating that *"employers were ... benevolent enough to provide that support"* |  | *"On an organizational level, the debriefing would help to generate a therapeutic climate where cognitive, affective, and behavioral symptoms can be openly discussed"* |
| Brandt et al. (2009) |  | Non-participation may have been due to *"logistical reasons"* specific to the studied population (hospital personnel). | Participants reported how important it was to have the support of managers or departmental chiefs. | *"Ongoing communication is critical in the disaster environment. Individual and group isolation must be avoided."* | *"Sharing the experience… in debriefing groups appeared to help people move from distancing to integrating their inner experience with the outside world"* |
| Carlier et al. (1998) | *"The formal seven-phase CISD procedure was adhered to in the debriefing"* | *"for operational reasons about half of the officers involved failed to undergo debriefing"* | The study/intervention was commissioned but not run by the organisation. |  | Participants *"perceived debriefing to be helpful"* even though *"it did not influence their emotional adaptation"* |
| Carlier et al. (2000) | *"The debriefing adhered to the structure"* laid out by the original authors. | *"Randomization was not feasible"* due to organisation regulations.   The model was *"adapted for use with individual trauma victim"* | Debriefing is offered as *"standard practice"* in the outlined organisation. | There is a *"natural tendency in people who have recently been traumatized to seek emotional support"* from others. | *"Respondents who received debriefing generally expressed great satisfaction with it"* but there was no relationship between satisfaction and symptom levels. |
| Chemtob et al. (1997) | *"The treatment in this study may well have diverged from others that may be more or less effective."* | The authors highlight that it is *"not readily feasible in a postdisaster situation to withhold treatment from a group"* |  | The *"didactic component to our debriefing protocol was very helpful to the participants"* | The results of the study suggested that there were *"high levels of client satisfaction"* but those had *"no differential impact on psychometric measures"* |
| Chongruksa et al. (2012) |  | Some officers could not be included in the intervention following transferral to another province. | Participants expressed that one of their top stressors was treatment from commanding officers and scarcity of organisational resources. | The intervention sought, as a primary aim, to *"reduce group resistance and build cohesion".* Seeing others impart their stories helped participants to open up. |  |
| Chongruksa et al. (2015) |  | Barriers to help seeking in this population include *"fears of public stigma and the character of military culture which allows seeking help only from a comrade"* | The paper recognises the importance of supervisors for assessing work-related outcomes; job performance and worker retention. | The reductions in symptom severity were suggested to arise from the social support that was provided by the other members of the intervention group. | *"participants expressed their appreciation…*[for the opportunity] *to express their distress and concerns regarding deployment with rangers from different units"* |
| Cigrang et al. (2005) | The intervention was limited to provide only psychological first aid, and specifically removed single session debriefing. The sessions were also shorter than standard procedure. | The *"risk is omnipresent"* in a way that is unique to that workplace context Threats to survival were *"very likely"* at all times. | Referrals to treatment were *"facilitated by unit leadership".* Managers were a part of decision making during the assessment and recommendation sessions. | Participants reported that recovery was bolstered by being *"well-connected and supported by his military peers"* and generally high levels of social support in their environment. | The similarity of the debriefing to operational debriefings meant soldiers could participate without stigma. Working with managers was also seen to be beneficial to recovery. |
| Cigrang et al. (2017) | Researchers adapted previous models of mental health provision to deliver shorter sessions, thereby overcoming logisitical issues specific to the organisational context. | The barriers for help-seeking in this organisation incl. stigma, career impact, distrust of mental health services, and the logistics of attending multiple sessions. | The intervention was facilitated by the organisation and located within the organisation's own treatment facilities. |  | Classification of the intervention as primary care, rather than as a mental health appointment helped to reduce perceived stigma. |
| Deahl et al. (1994) | The intervention *"was not rigorously standardised…*[for] *content and timing"* but *"contained the same common elements"*. | The cultural context *"emphasises tough-mindedness and tends to militate against seeking help for psychological problems."* | *"Organisational and managerial"* factors were highlighted among the curative factors in recovering from exposure. *"training was, by operational necessity"* | In this study, *"group cohesion factors"* appeared to have "*no beneficial effect"* | *"many soldiers valued the opportunity to express feelings of anger and guilt and derived comfort from the realisation that these were a normal emotional response to trauma"* |
| Deahl et al. (2000) | The intervention followed a manualised protocol. | The first session was tailored to discuss the types of *"events* [servicemen] *were likely to encounter".* Operational factors meant some could not participate. | Debriefing was delivered as part of the organisation's routine operational training package. Commanders were involved in allocating to condition. | Recognises the *"complex group dynamics which develops amongst a cohesive close-knit group who have shared a common trauma"* | Debriefing *"may have a beneficial effect*[s]*"* not captured by existing outcome measures and thus additional ones are needed e.g. substance misuse. |
| Dickstein et al. (2013) |  | Participants received a version of the intervention that was adapted for use in military populations. | The intervention was delivered as part of routine clinical care. |  |  |
| Difede et al. (2007) | The intervention was based on a manualised protocol. Sessions were assessed for adherence. | The protocol was *"modified for use with disaster workers".* *"Some patients had difficulty attending sessions due to logistical constraints".* | The intervention was conducted by a external research team. |  |  |
| Drury et al. (2013) |  | Participants identified stressors unique to emergency response: including lack of down-time, actions of managers and the ‘target culture’. | There was some disagreement on the effectiveness of support provided by line managers or the organisation. | Participants agreed that services would be improved through additional: *"peer support programmes... and other techniques for peer supporters"* |  |
| Firing et al. (2015) | The intervention was developed to utilise the benefits from traditional debriefing and psychological debriefing, following guidelines from an operational handbook. | The model was developed to cater to the needs of emergency responders. | Participants found it helpful to have the support from their commanders/ managers: *"support from the commander created room for reflection and ... a safe learning climate"* | Participants expressed the benefits of *"knowing that the others* [were] *also affected by strong impressions"* it helped to normalise responses and create a "*kinship*" | Participants stated that *"putting the impressions ... into words* [was] *a restoration process".* The sessions also helped participants to *"put into words"* the experience. |
| Frappell-Cooke et al. (2010) | The intervention closely followed the guidelines laid out in previously published work. | Has a *"distinctive organizational culture".* E.g. Stigma is a likely barrier to help-seeking in high-threat organisations. | The authors of the research suggest that the intervention acts as reassurance that the organisation has in place a protective system for its workers. | *"those suffering higher levels of trauma-related stress perceived less social support from other unit members and less of a support network during the tour"* |  |
| Gould et al. (2007) | The intervention referred to a longstanding programme of support using TRIM. | The military reflects *“a highly selected group and unrepresentative of the civilian populations being trained to fight and work in a cohesive group”* | Managers/leaders were supportive of participation; were involved in allocation and assessment of personnel to the programme. | Greater understanding achieved *"through exposure to other peoples’ related experiences."* *"most personnel turned to informal networks for support"* (i.e. peers) | The authors suggest that *"morale may have account*[ed] *for the"* changes in symptom scores. As well as the differences in *"emotional and practical skills".* |
| Greenberg et al. (2010) | The intervention was delivered by *"experienced service personnel who have been trained* [and assessed] *by military mental health professionals".* | Lower response numbers due to *"drafting policies"* than nonresponse. | *"Ministry of Defence has been keen to explore the development of occupational interventions".* Soldiers encouraged to *“exercise”* the system in place. | The intervention *"capitalize*[s] *on the social cohesion available within military units"* and "*aims to facilitate peer and unit support"* |  |
| Grundlingh et al. (2017) | The current study was conducted *"with consideration for the parameters suggested by CISD proponents".* | The intervention was *"tailored to violence researcher needs"* and a portion drawn from CISD techniques. | *"Perceived organisational support was strongly associated with those researchers who reported lower levels of end-line emotional distress"* | Positive associations with support from family and friends indicate *"that those with higher levels of distress were more likely to draw upon social networks."* | *"Cultural norms … may have normalised the experience for these researchers and prevented significant emotional distress."* |
| Gunasingam et al. (2015) | The protocol was designed by a senior health professional for the study. | *"Long working hours, poor work–life balance and the inability to commit to personal and social activities … associated with burnout"* in junior doctors. | Authors highlight need to address the following issues at organisational level: *"lack of support from senior staff, and ambiguity of future career progression".* | Informal debriefing among colleagues were described as commonly employed tools *"to manage stress and prevent burnout".* | The debriefing sessions were *"considered a valuable support mechanism"* but did not improve burnout scores. |
| Halpern et al. (2009) |  | *"a context of an organizational culture that stigmatizes emotional vulnerability"* | Supervisors who were seen as supportive were described with "considerable appreciation" while those who were unsupportive were were described in "angry, resentful, and disappointed tones". Many distrusted *"whether management had their best interests in mind"* | Participants reported that *"knowing somebody cared enough to … connect"* or spending a timeout with peers was an important curative factor. | *"Acknowledgement of the incident as critical"* and  *"valuing the* [staff's] *work"* were among the factors perceived as beneficial during recovery. |
| Harris et al. (2011) | Authors highlight the need *"to describe the exact nature and length of the debriefing; qualifications, experience, and quality of the debriefers; and the timing of the debriefing"* | The vocation in question "*exposes men and women to distressing sights, sounds, smells, and memories".* Supportive organisational practices are crucial in this context. | The authors suggest that employee assistance programmes may be beneficial in protecting emergency personnel and should represent an occupational requirement. | Perceived social support from friends and colleagues were among the significant predictors of participants' mental health indexes. | *"emergency responders for the most part appreciate and are pleased with debriefings"* |
| Hunt et al. (2013) | The procedures were delivered using guidance provided by National Institute for Clinical Excellence |  | The intervention was facilitated by a member of staff and run during a working day to encourage attendance. "*the chief constable approved the evaluation process*" | *"it may have been helpful for some individuals to speak to a colleague about their experiences, since social support has been shown to be useful"* |  |
| Hutton et al. (2010) | The protocol was based on CISD but instead of emergency response, it was applied to deal with responses to a child patient's death. | Some listed threats to loss of professional integrity and expectations as barriers to help-seeking. | The authors note that *"Support from nursing leadership is essential for the success of this type of intervention."* | The *"most common strategy for paediatric nurses caring for dying children was to share their experiences with colleagues".  The most satisfying elements were team collaboration and the relationship with the family.* | *"Many evaluations noted how helpful it was to hear how other disciplines viewed what happened from their perspective"* |
| Jones et al. (2017) |  | During deployment mental health care opportunities are limited and perceived barriers to care, such as stigmatization, appears to be heightened. | *"TRiM is an acceptable intervention among military personnel as long as it is fully supported by military commanders"* |  |  |
| Kenardy et al. (1996) | *"there was no standardization of debriefing services ... thus we do not know to what extent the stress debriefing matched* [CISD]*"* | *"Participants may fear that ...attendance at stress debriefing will stigmatize them and that they will be perceived as weak by their colleagues"* | *"Professional organizations involved in helping may have had debriefing as an integral component of their standard procedures"* |  | *"Emergency service workers rated the debriefing as having considerable personal value"; as "Very" or "Extremely" helpful* |
| Leonard & Alison (1999) |  | *"the critical incident itself is less a feature of importance than the context within which it is received by the officer and those around him"* | Multiple officers commented on the lack of support offered by their department which significantly affected their ability to cope. | Officers *"used instrumental and emotional social support in conjunction with adaptive coping, focusing on and venting of emotions"* | Officers reported that the debrief gave their feelings meaning; made them believe the department cared; and helped to normalise their reactions. |
| Macnab et al. (1998) | *"Each of the sessions followed the recommendations of Mitchell"* | The authors suggest that stress debriefing may only work for the emergency services and the application in other contexts may be *"inappropriate"* | Formal debriefing was made available to all employees.  The authors highlight the need for *"psychological profile assessment during training"* | Participants *"used talking with a significant other, colleague, or friend"* as a method for managing stress. | The CISD team were perceived as unhelpful as the interest in staff welfare was seen as unusual for that organisation. |
| Macnab et al. (2004) | The intervention followed principles of CISD. Screening was assessed over the phone. | The authors suggest it is crucial to have *"support systems*" for emergency responders. That, they receive *"instruction on stress management"* as part of their training. | *"It was particularly crucial that the labour union ... was supportive of the study, as there always is the potential for distrust between the union and management"* |  |  |
| Matthews (1998) | The intervention was *"based on the Mitchell model"* | Carers and nurses are often assaulted by patients. As a result, staff in direct care settings are particularly at risk of developing symptoms of PTSD. | The intervention was discussed regularly at staff meetings to ensure staff were aware of its availability. Managers were contacted for identifying participants. | Participants shared their individual methods for coping to help each other establish a variety of strategies. | *"CISD is usually evaluated positively by health care and emergency service workers".* Over half felt that debriefing was helpful in reducing stress. |
| Mitchell, Stevenson, and Poole (2000) | *"Group debriefing is the most frequent form of CISD although often, individual debriefing is offered based on need and practicality"* | *"the wider context of work needs to be considered when examining the impact of critical incidents"* | *"Support and recognition by supervisors during and after a threatening incident is crucial to subsequent psychological resolution"* | *"Discussing the facts ... following an incident, or in a CISD, provides a setting in which points of view can be shared and social support given and received"* | *"Officers appear to derive some psychological benefit from structured discussion following an incident"* in spite of its minimal effects on symptoms |
| Robinson & Mitchell (1993) | The debrief follows the guidelines as outlined in previously published research. | *"the management of trauma … needs to bear in mind predisposing and contextual job factors which may prepare the individual to manage trauma at work"* |  | *"Felt a common bond between members at the session."* | Participants *"felt there was great value in the police and ambulance attending the debriefing, as we can all learn from one another"* |
| Palgi et al. (2012) | *"Due to the problems with the debriefing technique, an alternative intervention was suggested."* Emphasis was placed on preparing staff to deal with trauma. | Soldiers reported of multiple stressors that extended outside the conflict zone: *“Two wars, outside and at home”* |  | *“What mainly helps me is family and friends.”  “With my friends here* [in the unit] *there is a common language.”* | *“Everybody in that room became my brother or sister. The war blends/binds us together.”* |
| Regehr & Hill (2001) | The intervention used draws upon common elements from several models of psychological debriefing including *"a psycho-educational group meeting".* | Trauma *"in emergency responders have a profound effect on... emergency service organizations to continue to be responsive to the needs of the public"* | *"In times of cuts ... in many emergency service organizations, the perception that someone cares… may be important to ... well-being and job performance."* | *"The group modality allows for ventilation of feelings, encourages mutual aid within the organization and reinforces innate abilities to cope"* | *"Firefighters attending crisis debriefing groups felt that they were beneficial to them personally (86%) and assisted in reducing their level of stress (77%)"* |
| Rick et al. (2006) | The "*trauma care programme* [was] *adapted and developed in response to new latest research in the area"* | Trauma management procedures had notably *"ceased because of staff changes and the transfer of trained managers to other jobs (as part of larger organisation wide change)."* | *"those who felt more positive about the organisational support … following the traumatic incident also had significantly lower absence levels"* | *"social and emotional support delivered in an empathic manner is important in promoting recovery from PTSD****"*** | Participants reported a *"high degree of satisfaction with the support provided on the day of the incident"* which endured across time. *‘I felt the company cared about my well‐being’* |
| Ruck et al. (2013) | For this intervention, a *"form of debriefing based on the work of Mitchell and Everly (1996)"*, however was adapted for use in the Prison Service. | *"Prison Service workers are prone to develop trauma"* from exposure to assault, riots, and deaths. The context prohibits use of RCTs due to ethical and legal concerns. | *"It is reasonable to expect an employer to provide a safe environment for its employees, exposing staff to critical incidents without support could be viewed as negligent."* |  |  |
| Rudd et al. (2015) | *"To maintain treatment fidelity, feedback was provided...to ensure that the therapists adhered to the brief CBT manual; both therapists achieved .90% fidelity ratings."* | Treatment in a military environment offers a number of unique challenges... In these circumstances, two primary issues are flexibility and brief duration, both of which are essential for successful implementation within the high-tempo, fluid, and unpredictable military system. | The organisation was involved in the coordination of identifying participants and scheduling follow-up assessments. |  | *"effective treatment...does not require complete remission of symptom severity but rather the development of core skills in the areas of emotion regulation"* |
| Shalev et al. (1998) | The intervention followed a model previously used in the U.S. Army, but here applied to Israeli soldiers. | In the target population, there are a number of factors that may influence the intervention, for example the military rank of other participants. Thus military rank has to be suspended. | Commanding officers were involved in providing researchers with relevant information. Commanding officers also engaged participants with fact-finding debriefing beforehand. | *'debriefing essentially resulted in the "normalization" of the group i.e., increasing similarity among group members and minimizing outliers'* | *"effects may be attributable to enhancing group cohesion"  "sessions were followed by a sense of relief or a "spiritual purge."* |
| Shoval-Zuckerman et al. (2015) | The intervention *"was based on a military stress debriefing protocol".* | The current model was *"formulated after targeting the special needs of the reserve soldiers".* This includes processing separation from unit members as well as family. | *"group debriefing* [was] *consistent with the military tradition of after-action reviews".* The debriefing was co-delivered by the commander of the relevant military unit. | The intervention was *"based on the assumption that the military group provides a significant support network and source of strength."* | Participants felt the debriefing had helped restore them to optimal functioning despite not improvement on PTSD symptom severity. |
| Tehrani et al. (2001) | The authors highlight that *"assessment tools designed for use in hospitals or laboratory settings"* may inappropriate for use other workplaces. | The organisation was under pressure to *"manage the health and well-being of their staff* [and] *run the retail operation during… one of the busiest times of the year".* | Internal occupational health approaches benefit from manager involvement which aids in the recognition of pre-existing issues within an organisation, and assess workplace outcomes. | Employees said *"that it was reassuring to hear the accounts of others involved in helping the injured passengers describing their experiences and responses."* | Debriefing is *"valued by both debriefers and employees"* even though *"there is little evidence … to support it as an effective treatment".* |
| Tuckey & Scott (2014) | *"Each intervention was led by one of two consultant mental health professionals who were trained and highly experienced in using the techniques"* | *"There are important differences between workers...compared with the general population… Responders are aware of the danger and active in...managing the incident"* | The intervention was available through an internal employee assistance programme. Senior managers were involved in approval of the study. | *"social networks* [are] *thought to be very important as group factors, including the climate for support influence psychological health outcomes"* | *"CISD may meet other needs (e.g., connecting with peer support, providing a bridge to later treatment) and/or be beneficial for… well-being and quality of life"* |
| Waelde et al. (2017) | Participants completed a home study programme following a previously validated manual. The *"program was modified from its original format for use in disaster training."* | *"previous work* [indicates] *that disaster providers may themselves be in need of self-care as a result of direct and vicarious exposures to disaster stressors"* | The intervention comprised participants from different occupations and organisations, and was delivered by an external research team. |  | *"participants were very satisfied with the training and had high expectancies that this mindfulness program would be useful for both survivor and self-care"* |
| Wu et al. (2012) | *"The debriefings were based on the CISD protocol"* but were modified to include a *"cohesion training section"*. Protocol adherence was assessed by a rating system. | The model was developed to cater to *"the unique characteristics of Chinese military rescuers"* in particular; unit cohesion differentiates them from other emergency response. | While not part of standard training, the authors highlight the potential benefits of *"conducting and practicing such training at subsequent times"* e.g. during pre-deployment. | *"They have a strict hierarchy and each belongs to a specific squad, platoon and company. Therefore, the relationship...is much closer than other rescue groups"* |  |
| Young & Parr (2004) | *"Each treatment group included a peer officer who was trained in the CISM and CISD models"* | Some participants reported being *"mildly teased about their participations"* indicating a culture of stigma around help-seeking within the police forces. | The debriefing occurred between shifts, sometimes away from the department.  *"Police cadet training briefly covers posttraumatic stress ... but is limited in scope"* | *"Teaching occurred from officer to officer… This interaction seemed especially effective and helpful and also built a sense of group cohesion"* | Participants reported that *"mutual support was a highlight of this group experience".* |
|  |  |  |  |  |  |
|  |  |  |  |  |  |
|  |  |  |  |  |  |
|  |  |  |  |  |  |
